# The characteristics of average abundance function with mutation of multi-player threshold public goods evolutionary game model under redistribution mechanism

**DOI:** 10.1186/s12862-021-01847-0

**Published:** 2021-08-04

**Authors:** Ke Xia

**Affiliations:** School of Economics, Zhengzhou University of Aeronautics, Zhengzhou, China

**Keywords:** Evolutionary game, Average abundance function, Stochastic process, 91A22

## Abstract

**Background:**

In recent years, the average abundance function has attracted much attention as it reflects the degree of cooperation in the population. Then it is significant to analyse how average abundance functions can be increased to promote the proliferation of cooperative behaviour. However, further theoretical analysis for average abundance function with mutation under redistribution mechanism is still lacking. Furthermore, the theoretical basis for the corresponding numerical simulation is not sufficiently understood.

**Results:**

We have deduced the approximate expressions of average abundance function with mutation under redistribution mechanism on the basis of different levels of selection intensity $\omega$ (sufficiently small and large enough). In addition, we have analysed the influence of the size of group *d*, multiplication factor *r*, cost *c*, aspiration level $\alpha$ on average abundance function from both quantitative and qualitative aspects.

**Conclusions:**

(1) The approximate expression will become the linear equation related to selection intensity when $\omega$ is sufficiently small. (2) On one hand, approximation expression when $\omega$ is large enough is not available when *r* is small and *m* is large. On the other hand, this approximation expression will become more reliable when $\omega$ is larger. (3) On the basis of the expected payoff function $\pi \left( \centerdot \right)$ and function $h(i,\omega )$, the corresponding results for the effects of parameters (*d*,*r*,*c*,$\alpha$) on average abundance function $X_{A}(\omega )$ have been explained.

## Background

### Literature review for average abundance function

It is well known that aspiration rule reflects the characteristics of self-learning. Furthermore, individuals will compare their own payoff with aspiration level which reflects the concept of “satisfaction”. Generally speaking, the discussion on aspiration rule contains three parts: (1) the research on average abundance function under different mechanisms; (2) the research on average abundance function when aspiration level is a complex function; (3) the comparison between aspiration rule and imitation rule.

### The research on average abundance function under different mechanisms

On one hand, Zhang et al. [1] authenticated that suitable aspiration level can improve cooperative behavior and the evolution time series show the so-called “ping-pong effect” for aspiration levels. Chen et al. [2] demonstrated that average abundance function increases with payoff weight coefficient. Zeng et al. [3] corroborated that risk-adaptation mechanism based on aspiration level can make average abundance function reach the maximum value. Liu et al. [4] affirmed that the effects of aspiration level is non monotonic.

On the other hand, Perc et al. [5] affirmed that interactions among humans often involve group interactions, and they also involve a larger number of possible states even for the most simplified description of reality. Szolnoki et al. [6] demonstrated that a moderate fraction of cooperators can prevail even at very low multiplication factors if the critical mass is minimal.

It should be noted that Chen et al. [7] affirmed that the random WSLS mechanism and local weight coefficient can improve average abundance function. Perc et al. [8] analyzed the basic characteristics of public goods game, reviewed recent advances in the study of evolutionary dynamics of group interactions on top of structured populations and compared these results with those obtained on well-mixed populations. Liu et al. [9] corroborated that the modified WSLS mechanism based on aspiration level can promote average abundance function and this effect has nothing to do with the initial distribution.

In addition, Matsen et al. [10] demonstrated that WSLS mechanism can greatly improve average abundance function. Chen et al. [11] authenticated that moderate aspiration level can improve average abundance function. Du et al. [12] affirmed that the effect of redistribution on cooperative promotion under different aspiration distributions varies with the proportion of redistribution. Zhou et al. [13] investigated how heterogeneity in the rules for behavior updating alters the evolutionary outcome.

Moreover, Peng et al. [14] corroborated that the influence of the payoff of betrayal strategy on average abundance function can be ignored when migration mechanism is taken into account. Lin et al. [15] affirmed that the migration of individuals with low aspiration level can improve average abundance function. Yang et al. [16] authenticated that appropriate aspiration level can promote the migration of collaborators. In addition, this kind of migration promotes the formation of clusters and improves average abundance function.

### The research on average abundance function when aspiration level is a complex function

It is well known that aspiration level is not always fixed. Moreover, many scholars have studied the properties of average abundance function when aspiration level is a complex function. Furthermore, aspiration level of each stage is a complex function related to the corresponding coefficients of the previous stage. On the basis of this finding, it can be deduced that aspiration level is closely related to evolutionary time.

On one hand, Platkowski et al. [17] demonstrated that average abundance function decreases with the size of population. Platkowski et al. [18] affirmed that the critical condition to ensure the maximization of average abundance function is independent of initial distribution and group size when aspiration level is at an appropriate level. Feng et al. [19] corroborated that the cost can led to the bidirectional effects of aspiration rule on average abundance function. Tang et al. [20] authenticated that appropriate aspiration level improves average abundance function when cost-benefit ratio is at the suitable level. In addition, the suitable level is related to the maximum Laplacian eigenvalue of the network. Wu et al. [21] demonstrated that evolutionary outcomes under heterogeneous aspiration level is the same as those under homogeneous aspiration level.

On the other hand, Rong et al. [22] corroborated that low aspiration level promotes the coexistence of different strategies and appropriate aspiration level improves the average abundance function of generous strategy. Wu et al. [23] demonstrated that the effects of aspiration level on average abundance function under synchronous updating is different from that under asynchronous updating. Wakano et al. [24] affirmed that average abundance function becomes more stable when learning coefficient based on aspiration level increases. Platkowski et al. [25] affirmed that average abundance function increases when global aspiration level is taken into consideration. Roca et al. [26] corroborated that average abundance function increases with greediness. However, it decreases when greediness is too high.

### The comparison between aspiration rule and imitation rule

The essence of aspiration rule lies in the comparison between payoff and aspiration level. In addition, the essence of imitation rule lies in the comparison between own success and another individual’s success. It is well known that many scholars study the influence of different rules on evolutionary results based on the previous analysis.

On one hand, Du et al. [27] also demonstrated that the sustainable time of public resources under aspiration rule is longer than that under imitation rule. Zhang et al. [28] demonstrated that aspiration level leads to the negative feedback effect. In addition, aspiration level promotes the heterogeneity distribution of node degree. Du et al. [29] deduced the dominant condition for average abundance function under aspiration rule. Perc et al. [30] affirmed that heterogeneity in aspirations may be key for the sustainability of cooperation in structured populations.

On the other hand, Li et al. [31] demonstrated that high aspiration level leads to the disappearance of the coexistence of different strategies. In addition, average abundance function increases with interaction coefficient. Du et al. [32] affirmed that the effects of aspiration level on average abundance function in structured population under weak selection intensity is similar to that in well-mixed population. Li et al. [33] corroborated that the combination of aspiration rule and modified self-questioning mechanism leads to the increasing of average abundance function.

Moreover, Xu et al. [34] demonstrated that average abundance function reaches the highest value when both interaction coefficient and aspiration level are at appropriate level. Xuesong Liu [35] affirmed that the combination of aspiration rule and imitation rule is conducive to the increasing of average abundance function. Chen et al. [36] corroborated that the suboptimal selection based on aspiration level is beneficial for average abundance function.

### Main work

Firstly, we analyze the basic properties of the multi-player threshold public goods evolutionary game model under redistribution mechanism and we obtain the the intuitive expression of average abundance function with mutation. Secondly, we deduce the approximate expression of average abundance function when selection intensity is sufficient small and selection intensity is large enough. At last, we analyze the influence of different parameters on average abundance function.

## Methods

## Model for multi-player threshold public goods evolutionary game model

### The research on multi-player threshold public goods evolutionary game model

#### The multi-player evolutionary game model

In a finite well-mixed population of size *N*, *i* is the number of *A* players. In this population, groups of size *d* are assembled randomly. The focal player can be of type *A*, or *B*, and encounter a group containing *k* other players of type *A*, to receive the pay-off ${a}_{k}$ or ${b}_{k}$. The multi-player game [37–49] payoff matrix is defined as Table 1.

**Table 1:** The multi-player game payoff matrix

	$d-1$	...	*k*	...	0
*A*	${{a}_{d-1}}$	...	${{a}_{k}}$	...	${{a}_{0}}$
*B*	${{b}_{d-1}}$	...	${{b}_{k}}$	...	${{b}_{0}}$

The expected payoff for any *A*, or *B* in a population of size *N*, with *i* players of type *A* and $(N-i)$ players of type *B*, are defined by ${{\pi }_{A}}(i)$ and ${{\pi }_{B}}(i)$. Based on Table 1, the general expression of expected payoff function can be defined as follows:

1a $$\begin{aligned} {{\pi }_{A}}(i)= & {} \sum \limits _{k=0}^{d-1}{{{P}_{A}}\left( k,d;i,N \right) {{a}_{k}}}=\sum \limits _{k=0}^{d-1}{\frac{C_{i-1}^{k}C_{N-i}^{d-1-k}}{C_{N-1}^{d-1}}{{a}_{k}}} \end{aligned}$$

1b $$\begin{aligned} {{\pi }_{B}}(i)= & {} \sum \limits _{k=0}^{d-1}{{{P}_{B}}\left( k,d;i,N \right) {{b}_{k}}}=\sum \limits _{k=0}^{d-1}{\frac{C_{i}^{k}C_{N-i-1}^{d-1-k}}{C_{N-1}^{d-1}}{{b}_{k}}} \end{aligned}$$

#### The multi-player threshold public goods evolutionary game model under redistribution mechanism

It is well known that *c* is an initial endowment given to every individual. The cooperator will provide *c* to the common pot. If the number of cooperators is smaller than *m*, all pay-offs will be zero. In addition, if the number of cooperators is not smaller than *m*, the total amount of funds in the common pool will be multiplied by the multiplication factor *r* [50–56] and it will be shared equally for all members. Based on the above discussions, the expressions of the corresponding matrix elements ${{a}_{k}}$ and ${{b}_{k}}$ in Table 1 can be obtained:

1c $$a_{k} \begin{array}{*{20}c} {\begin{array}{*{20}c} = \\ \end{array} } \\ \end{array} \left\{ {\begin{array}{*{20}c} 0 & {k < m - 1} \\ {\frac{{k + 1}}{d}rc} & {k \ge m - 1} \\ \end{array} } \right.\left( {m \ge 1} \right){\text{ }}$$

1d $$b_{k} {\text{ = }}\left\{ {\begin{array}{*{20}c} 0 & {k < m} \\ {\frac{k}{d}rc + c} & {k \ge m} \\ \end{array} } \right.\left( {m \ge 1} \right){\text{ }}$$

Furthermore, a redistribution mechanism [57–61] is introduced into this model. At a fixed proportion $\tau$, every individual in the group is required to hand in part of payoff. In addition, this kind of compulsory expenditure is called second-order payment. The second-order payment of every individual in the group will be aggregated and it will be redistributed to everyone in the group uniformly.The expressions of the corresponding matrix elements ${{a}_{k}}$ and ${{b}_{k}}$ in Table 1 can be easily deduced based on the above discussions:

2 $$\;a_{k} \begin{array}{*{20}c} {\begin{array}{*{20}c} = \\ \end{array} } \\ \end{array} \left\{ {\begin{array}{*{20}c} 0 & {k < m - 1} \\ {\frac{{k + 1}}{d}\left( {r - \tau } \right)c + \tau c} & {k \ge m - 1} \\ \end{array} } \right.$$

3 $$b_{k} \begin{array}{*{20}c} {\begin{array}{*{20}c} = \\ \end{array} } \\ \end{array} \left\{ {\begin{array}{*{20}c} 0 & {k < m} \\ {\frac{{k + 1}}{d}\left( {\tau - \tau } \right)c + c} & {k \ge m} \\ \end{array} } \right.$$

### The research on average abundance function with mutation

#### Game behaviors of aspiration dynamics

The game behavior has been studied by many scholars [62–67]. Furthermore, the transition probability equation of the evolutionary process can be deduced based on above analysis:

4 $$\begin{aligned} T_{i}^{+}= & {} \left( 1-\delta \right) \frac{N-i}{N}\frac{1}{1+{{e}^{-\omega (\alpha -{{\pi }_{B}}(i))}}}+\delta \frac{N-i}{N} \end{aligned}$$

5 $$\begin{aligned} T_{i}^{-}= & {} \left( 1-\delta \right) \frac{i}{N}\frac{1}{1+{{e}^{-\omega (\alpha -{{\pi }_{A}}(i))}}}+\delta \frac{i}{N} \end{aligned}$$

6 $$\begin{aligned} T_{i}^{0}= & {} 1-T_{i}^{+}-T_{i}^{-} \end{aligned}$$

The $\omega$ represents selection intensity [68, 69] and $\alpha$ represents aspiration level which reflects the satisfaction of individuals in formulas (4)–(6).

#### The characteristics of average abundance function with mutation

The proportion of individuals choosing strategy *A* (which can be defined as $\frac{j}{N}$) in the population is a random variable based on the transition probability equation defined by formulas (4)–(6). In addition, the probability distribution of $\frac{j}{N}$ can be defined as ${{\vartheta }_{j}}(\omega )$. Thus, the expected value of the proportion of individuals choosing strategy *A* [70–73] in the population is called the average abundance function ${{X}_{A}}(\omega )$:

7 $$\begin{aligned} {{X}_{A}}(\omega )=\sum \limits _{j=0}^{N}{\frac{j}{N}}{{\vartheta }_{j}}(\omega ) \end{aligned}$$

Furthermore, the intuitive expression of the average abundance function can be obtained through theoretical deduction when the detail balance condition is taken as the breakthrough point.

**Proposition 2-2**    When the transition probability equation of evolutionary state is defined by formulas (4)–(6), the intuitive expression of average abundance function is as follows:

7a $$\begin{aligned} X_{A}(\omega )= & {} {\frac{1}{N}}{\frac{\sum \limits _{j=1}^{N}{j{\prod \limits _{i=0}^{j-1}{h(i,\omega )}}}}{1+\sum \limits _{j=1}^{N}{\prod \limits _{i=0}^{j-1}{h(i,\omega )}}}} \end{aligned}$$

7b $$\begin{aligned} h\left( i,\omega \right)= & {} \frac{N-i}{i+1}\times \frac{1+{{e}^{-\omega (\alpha -{{\pi }_{A}}(i+1))}}}{1+{{e}^{-\omega (\alpha -{{\pi }_{B}}(i))}}}\times \frac{1+\delta {{e}^{-\omega (\alpha -{{\pi }_{B}}(i))}}}{1+\delta {{e}^{-\omega (\alpha -{{\pi }_{A}}(i+1))}}} \end{aligned}$$

Based on formula (1a), the expression of ${{\pi }_{A}}(i+1)$ and ${{\pi }_{B}}(i)$ in formula (7b) can be easily obtained:

7c $$\begin{aligned} {{\pi }_{A}}(i+1)= & {} \sum \limits _{k=0}^{d-1}{\frac{C_{i}^{k}C_{N-i-1}^{d-1-k}}{C_{N-1}^{d-1}}{{a}_{k}}} \end{aligned}$$

7d $$\begin{aligned} {{\pi }_{B}}(i)= & {} \sum \limits _{k=0}^{d-1}{\frac{C_{i}^{k}C_{N-i-1}^{d-1-k}}{C_{N-1}^{d-1}}{{b}_{k}}} \end{aligned}$$

The deduction process of formula (7a) can be found in related article [74]. On the whole, the establishment of formula (7a) lays a foundation for further analytical analysis and numerical simulation.

## Results

### The approximate expression of average abundance function when selection intensity is sufficient small

**Proposition 3-1**    The approximate expression of average abundance function with mutation of multi-player threshold public goods evolutionary game model under redistribution mechanism when selection intensity is sufficient small can be defined as follows:

8 $$\begin{aligned} {{X}_{A}}(\omega )= & {} \frac{1}{2}+\omega \frac{{1 - \delta }}{{1 + \delta }} \frac{1}{{{2}^{d+2}}}\frac{c}{d}\left[ \left( \frac{m\left( r-\tau \right) +d\tau }{r-\tau +d\tau }C_{d-1}^{m-1}+\sum \limits _{k=m}^{d-1}{C_{d-1}^{k}} \right) \left( r-\tau +d\tau \right) \right. \nonumber \\&\quad \left. -d\sum \limits _{k=m}^{d-1}{C_{d-1}^{k}} \right] \end{aligned}$$

#### Proof

Many scholars [75] have deduced the approximation expression of average abundance function when $\omega \rightarrow 0$:

9 $$\begin{aligned} {{X}_{A}}(\omega )=\frac{1}{2}+\omega \frac{{1 - \delta }}{{1 + \delta }} \frac{1}{{{2}^{d+2}}}\sum \limits _{k=0}^{d-1}{C_{d-1}^{k}}\left( {{a}_{k}}-{{b}_{k}} \right) \end{aligned}$$

By inserting formulas (2)–(3) into formula (9), we can get:

10 $$\begin{aligned} {{X}_{A}}(\omega )&=\frac{1}{2}+\omega \frac{{1 - \delta }}{{1 + \delta }} \frac{1}{{{2}^{d+2}}}\sum \limits _{k=0}^{d-1}{C_{d-1}^{k}}\left( {{a}_{k}}-{{b}_{k}} \right) =\frac{1}{2}+\omega \frac{{1 - \delta }}{{1 + \delta }} \frac{1}{{{2}^{d+2}}}\times \\&\qquad \left[ \sum \limits _{k=0}^{m-2}{C_{d-1}^{k}\times 0}+\sum \limits _{k=m-1}^{m-1}{C_{d-1}^{k}\left( \frac{k+1}{d}\left( r-\tau \right) c+\tau c \right) } \right. \\&\quad \left. +\sum \limits _{k=m}^{d-1}{C_{d-1}^{k}\left( \frac{k+1}{d}\left( r-\tau \right) c+\tau c-\frac{k}{d}\left( r-\tau \right) c-c \right) } \right] \\&=\frac{1}{2}+\omega \frac{{1 - \delta }}{{1 + \delta }} \frac{1}{{{2}^{d+2}}}\left[ C_{d-1}^{m-1}\left( \frac{m}{d}\left( r-\tau \right) c +\tau c \right) \right. \\&\left. \qquad +\sum \limits _{k=m}^{d-1}{C_{d-1}^{k}\left( \frac{1}{d}\left( r-\tau \right) c+\tau c-c \right) } \right] \\&=\frac{1}{2}+\omega \frac{{1 - \delta }}{{1 + \delta }} \frac{1}{{{2}^{d+2}}}c\left[ C_{d-1}^{m-1}\left( \frac{m}{d}\left( r-\tau \right) +\tau \right) \right. \\&\left. \qquad +\sum \limits _{k=m}^{d-1}{C_{d-1}^{k}\left( \frac{1}{d}\left( r-\tau \right) +\tau -1 \right) } \right] \\&=\frac{1}{2}+\omega \frac{{1 - \delta }}{{1 + \delta }} \frac{1}{{{2}^{d+2}}}c\left[ C_{d-1}^{m-1}\left( \frac{m}{d}\left( r-\tau \right) +\tau \right) \right. \\&\left. \qquad +\sum \limits _{k=m}^{d-1}{C_{d-1}^{k}\left( \frac{r}{d}-\frac{\tau }{d}+\tau \right) -\sum \limits _{k=m}^{d-1}{C_{d-1}^{k}}} \right] \\&=\frac{1}{2}+\omega \frac{{1 - \delta }}{{1 + \delta }} \frac{1}{{{2}^{d+2}}}c\left[ \frac{1}{d}\left( m\left( r-\tau \right) +d\tau \right) C_{d-1}^{m-1} \right. \\&\left. \qquad + \frac{1}{d}\left( r-\tau +d\tau \right) \sum \limits _{k=m}^{d-1}{C_{d-1}^{k}-\sum \limits _{k=m}^{d-1}{C_{d-1}^{k}}} \right] \\&=\frac{1}{2}+\omega \frac{{1 - \delta }}{{1 + \delta }} \frac{1}{{{2}^{d+2}}}\frac{c}{d}\left[ \left( \frac{m\left( r-\tau \right) +d\tau }{r-\tau +d\tau }C_{d-1}^{m-1}+\sum \limits _{k=m}^{d-1}{C_{d-1}^{k}} \right) \left( r-\tau +d\tau \right) \right. \\&\left. \qquad -d\sum \limits _{k=m}^{d-1}{C_{d-1}^{k}} \right] \\ \end{aligned}$$

Based on the discussions above, we can obtain formula (8). $\square$

In addition, formula (8) will play a significant role when analyzing the results of the numerical simulation.

## The approximate expression of average abundance function when selection intensity is large

We have already deduced the approximation expression of average abundance function with mutation when selection intensity is sufficient small. Naturally, how to obtain the corresponding approximation expression when selection intensity is large becomes a problem worthy of attention. In this section, the approximate expression of average abundance function when the selection intensity is large will be obtained based on the characteristics of expected payoff function ${{\pi }_{A}}(i+1)$ and ${{\pi }_{B}}(i)$. Furthermore, we will have a deeper understanding of the properties of average abundance function with mutation when selection intensity is large based on this approximation expression.

It can be deduced from formula (7a) that the main difficulty in obtaining the approximate expression of average abundance function is how to simplify the $\frac{1+\delta {{e}^{-\omega (\alpha -{{\pi }_{B}}(i))}}}{1+\delta {{e}^{-\omega (\alpha -{{\pi }_{A}}(i+1))}}}$ in function $h(i,\omega )$. In the beginning, by inserting formulas (2)–(3) into formula (1a), it can be deduced that:

10a $$\begin{aligned} {{\pi }_{A}}(i+1)= & {} \sum \limits _{k=m-1}^{d-1}{\frac{C_{i}^{k}C_{N-i-1}^{d-1-k}}{C_{N-1}^{d-1}}\left( \frac{k+1}{d}(r-\tau )c+\tau c \right) } \end{aligned}$$

10b $$\begin{aligned} {{\pi }_{B}}(i)= & {} \sum \limits _{k=m}^{d-1}{\frac{C_{i}^{k}C_{N-i-1}^{d-1-k}}{C_{N-1}^{d-1}}\left( \frac{k}{d}(r-\tau )c+c \right) } \end{aligned}$$

Then, if both ${{\pi }_{A}}(i+1)$ and ${{\pi }_{B}}(i)$ increase to very high level, the following inequalities will be established:

11 $$\begin{aligned} {e^{ - \omega (\alpha - {\pi _A}(i + 1))}}\gg & {} 1, {e^{ - \omega (\alpha - {\pi _B}(i))}} \gg 1 \end{aligned}$$

12 $$\begin{aligned} \delta {e^{ - \omega (\alpha - {\pi _A}(i + 1))}}> & {} 1,\delta {e^{ - \omega (\alpha - {\pi _B}(i))}} > 1 \end{aligned}$$

In addition, because of the establishment of formula (11) and (12), function $h\left( i,\omega \right)$ will gradually degenerate to the following expression:

13 $$\begin{aligned} h\left( {i,\omega } \right)\approx & {} \frac{{N - i}}{{i + 1}} \times \frac{{{e^{ - \omega \left( {\alpha - {\pi _A}\left( {i + 1} \right) } \right) }}}}{{{e^{ - \omega \left( {\alpha - {\pi _B}\left( i \right) } \right) }}}} \times \frac{{1 + \delta {e^{ - \omega \left( {\alpha - {\pi _B}\left( i \right) } \right) }}}}{{1 + \delta {e^{ - \omega \left( {\alpha - {\pi _A}\left( {i + 1} \right) } \right) }}}}\nonumber \\= & {} \frac{{N - i}}{{i + 1}} \times \frac{{1 + \delta {e^{ - \omega \left( {\alpha - {\pi _B}\left( i \right) } \right) }}}}{{1 + \delta {e^{ - \omega \left( {\alpha - {\pi _A}\left( {i + 1} \right) } \right) }}}} \times {e^{ - \omega \left( {{\pi _B}\left( i \right) - {\pi _A}\left( {i + 1} \right) } \right) }} \end{aligned}$$

By substituting formula (13) into formula (7a), the following proposition can be obtained:

**Proposition 4-1**  When both formula (11) and (12) are available, the approximate expression of average abundance function with mutation is as follows:

13a $$\begin{aligned} X_{A}(\omega )= & {} {\frac{1}{N}}{\frac{\sum \limits _{j=1}^{N}{j{\prod \limits _{i=0}^{j-1}{h_a(i,\omega )}}}}{1+\sum \limits _{j=1}^{N}{\prod \limits _{i=0}^{j-1}{h_a(i,\omega )}}}} \end{aligned}$$

13b $$\begin{aligned} {h_a}\left( {i,\omega } \right)= & {} \frac{{N - i}}{{i + 1}} \times \frac{{1 + \delta {e^{ - \omega \left( {\alpha - {\pi _B}\left( i \right) } \right) }}}}{{1 + \delta {e^{ - \omega \left( {\alpha - {\pi _A}\left( {i + 1} \right) } \right) }}}} \times {e^ {- \omega \left( {\pi _{B} \left( i \right) - \pi _{A} \left( {i + 1} \right)} \right) }} \end{aligned}$$

It can be deduced that the foremost factor of approximate expression of average abundance function with mutation is function ${h_a}\left( {i,\omega } \right) = \frac{{N - i}}{{i + 1}} \times \frac{{1 + \delta {e^{ - \omega \left( {\alpha - {\pi _B}\left( i \right) } \right) }}}}{{1 + \delta {e^{ - \omega \left( {\alpha - {\pi _A}\left( {i + 1} \right) } \right) }}}} \times {e^{ - \omega \left( {{\pi _B}\left( i \right) - {\pi _A}\left( {i + 1} \right) } \right) }}$. Furthermore, function ${h_a}\left( {i,\omega } \right)$ can be divided into three parts. The first part $\frac{N-i}{i+1}$ essentially reflects that individuals are in neutral drift state. The second part $\frac{1+\delta {{e}^{-\omega (\alpha -{{\pi }_{B}}(i))}}}{1+\delta {{e}^{-\omega (\alpha -{{\pi }_{A}}(i+1))}}}$ is the inference term caused by mutation. The third part ${e^{ - \omega \left( {{\pi _B}\left( i \right) - {\pi _A}\left( {i + 1} \right) } \right) }}$ reflects the comparison between ${{\pi }_{A}}(i+1)$ and ${{\pi }_{B}}(i)$.

## Verification the practicability of approximate expression (13a)

The approximate expression of average abundance function with mutation is defined as formula (13a) in proposition 4-1. Furthermore, we will verify the practicability of approximate expression (13a) in this section. It is worth noting that *r* and *m* will play an important role in determining whether strategy *A* is favoured by selection (${{X}_{A}}(\omega )>1/2$) or not (${{X}_{A}}(\omega )<1/2$). In addition, the practicability of approximate expression is closely related to whether ${{X}_{A}}(\omega )>1/2$ or ${{X}_{A}}(\omega )<1/2$. Therefore, the *r* and *m* should be taken into consideration carefully.

In the beginning, we have already deduced that the approximation expression of average abundance function when $\omega \rightarrow 0$ can be defined as follows:

14 $$\begin{aligned} {{X}_{A}}(\omega )= & {} \frac{1}{2}+\omega \frac{{1 - \delta }}{{1 + \delta }} \frac{1}{{{2}^{d+2}}}\frac{c}{d}\left[ \left( \frac{m\left( r-\tau \right) +d\tau }{r-\tau +d\tau }C_{d-1}^{m-1}+\sum \limits _{k=m}^{d-1}{C_{d-1}^{k}} \right) \left( r-\tau +d\tau \right) \right. \nonumber \\&\left. -d\sum \limits _{k=m}^{d-1}{C_{d-1}^{k}} \right] \end{aligned}$$

Based on formula (14), it can be deduced that the critical condition should satisfy:

15 $$\begin{aligned} {{X}_{A}}(\omega )= & {} \frac{1}{2}+\omega \frac{{1 - \delta }}{{1 + \delta }} \frac{1}{{{2}^{d+2}}}\frac{c}{d}\left[ \left( \frac{m\left( r-\tau \right) +d\tau }{r-\tau +d\tau }C_{d-1}^{m-1}+\sum \limits _{k=m}^{d-1}{C_{d-1}^{k}} \right) \left( r-\tau +d\tau \right) \right. \nonumber \\&\left. -d\sum \limits _{k=m}^{d-1}{C_{d-1}^{k}} \right] =\frac{1}{2} \end{aligned}$$

Then, on the basis of formula (15), the critical condition is defined as follows:

16 $$\begin{aligned} r = \frac{{\left( {m - d} \right) \tau C_{d - 1}^{m - 1} + \left( {\tau - d\tau + d} \right) \sum \limits _{k = m}^{d - 1} {C_{d - 1}^k} }}{{mC_{d - 1}^{m - 1} + \sum \limits _{k = m}^{d - 1} {C_{d - 1}^k} }} \end{aligned}$$

**Fig. 1:**
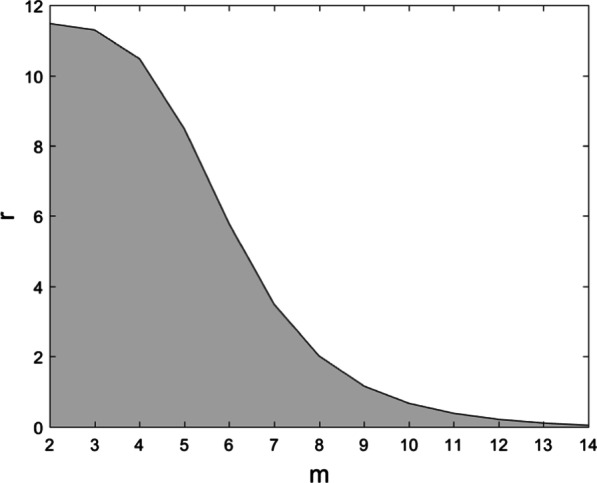
The relationship between *r* and *m* when considering whether strategy *A* is favoured by selection or not ($d=15,\tau =0.25$)

Moreover, it can be deduced that strategy *A* is favoured by selection (${{X}_{A}}(\omega )>1/2$) if $r>\frac{{\left( {m - d} \right) \tau C_{d - 1}^{m - 1} + \left( {\tau - d\tau + d} \right) \sum \limits _{k = m}^{d - 1} {C_{d - 1}^k} }}{{mC_{d - 1}^{m - 1} + \sum \limits _{k = m}^{d - 1} {C_{d - 1}^k} }}$. Then the curves describing the relationship between *r* and *m* based on formula (16) will be obtained (Fig. 1). In this condition, we set $d=15$. It can be seen from Fig. 1 that strategy *A* is not favoured by selection if *r* and *m* are in the shaded area of Fig. 1. Otherwise, strategy *A* is favoured by selection. Then the corresponding analysis can be carried out based on whether strategy *A* is favoured by selection or not.

### Strategy *A* is favoured by selection

#### Small *r* and large *m*

On the basis of formula (16) and Figure 5-1, it can be deduced that there are two conditions to ensure that strategy *A* is favoured by selection (${{X}_{A}}(\omega )>1/2$). The first condition is small *r* and large *m*. The second condition is medium *r* and medium *m*. Both conditions must satisfy the premise that $r>\frac{{\left( {m - d} \right) \tau C_{d - 1}^{m - 1} + \left( {\tau - d\tau + d} \right) \sum \limits _{k = m}^{d - 1} {C_{d - 1}^k} }}{{mC_{d - 1}^{m - 1} + \sum \limits _{k = m}^{d - 1} {C_{d - 1}^k} }}$.

**Fig. 2:**
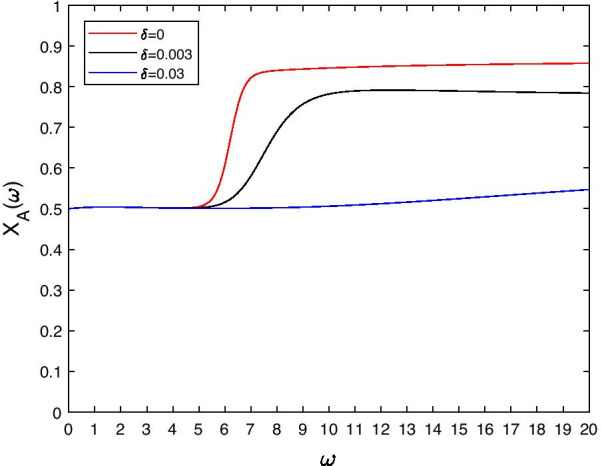
Average abundance function ${{X}_{A}}(\omega )$ changes with selection intensity $\omega$ under different mutations $\delta$ ($d=15,r=3,c=1,\tau =0.25,\alpha =1,m=12,N=100$) ($\delta =0,0.003,0.03$)

At this time the first condition is considered. In the beginning, we set the basic parameters as: $d=15$, $r=3$, $c=1$, $\tau =0.25$, $\alpha =1$, $m=12$, $N=100$. It should be noted that $r=3>\frac{{\left( {m - d} \right) \tau C_{d - 1}^{m - 1} + \left( {\tau - d\tau + d} \right) \sum \limits _{k = m}^{d - 1} {C_{d - 1}^k} }}{{mC_{d - 1}^{m - 1} + \sum \limits _{k = m}^{d - 1} {C_{d - 1}^k} }}=0.21$. Then, we can obtain the curves (Fig. 2) describing how average abundance function changes with selection intensity $\omega$ under different mutations $\delta$ ($\delta =0$, $\delta =0.003$ or $\delta =0.03$). On the basis of Fig. 2 , two corollaries can be obtained:

##### **Corollary 4.1.1**

*At first, average abundance function remains unchanged. Then, it increases. Finally, it remains unchanged when* $\delta =0$ *or* $\delta =0.003$.

##### **Corollary 4.1.2**

*At first, average abundance function remains unchanged. Then, it increases very slowly when* $\delta =0.03$.

On the basis of two corollaries, it can be deduced that approximation formula (13a) is insignificance when $\delta =0.03$. Then one property can be given as follows:

##### **Property 4.1**

*The approximation formula (*13a*) is meaningless if r is small, m is large, *, $r>\frac{{\left( {m - d} \right) \tau C_{d - 1}^{m - 1} + \left( {\tau - d\tau + d} \right) \sum \limits _{k = m}^{d - 1} {C_{d - 1}^k} }}{{mC_{d - 1}^{m - 1} + \sum \limits _{k = m}^{d - 1} {C_{d - 1}^k} }}$, *and* $\delta =0.03$.

#### Medium *r* and medium *m*

**Fig. 3:**
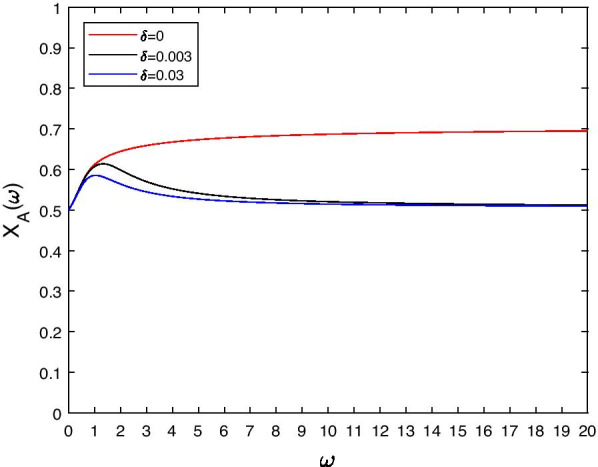
Average abundance function ${{X}_{A}}(\omega )$ changes with selection intensity $\omega$ under different mutations $\delta$ ($d=15,r=8,c=1,\tau =0.25,\alpha =1,m=8,N=100$) ($\delta =0,0.003,0.03$)

At present the second condition is taken into account. In the beginning, we set the basic parameters as: $d=15$, $r=8$, $c=1$, $\tau =0.25$, $\alpha =1$, $m=8$, $N=100$. It should be noted that $r=8>\frac{{\left( {m - d} \right) \tau C_{d - 1}^{m - 1} + \left( {\tau - d\tau + d} \right) \sum \limits _{k = m}^{d - 1} {C_{d - 1}^k} }}{{mC_{d - 1}^{m - 1} + \sum \limits _{k = m}^{d - 1} {C_{d - 1}^k} }}=2.02$. Then, we can obtain the curves (Fig. 3) describing how average abundance function changes with selection intensity $\omega$ under different mutations $\delta$ ($\delta =0$, $\delta =0.003$ or $\delta =0.03$). On the basis of Fig. 3, two corollaries can be obtained:

##### **Corollary 4.1.3**

*At first, average abundance function increases rapidly. Then, it basically remains unchanged when* $\delta =0$.

##### **Corollary 4.1.4**

*At first, average abundance function increases. Then, it decreases. Finally, it remains unchanged when* $\delta =0.003$ or $\delta =0.03$.

On the basis of two corollaries, it can be deduced that approximation formula (13a) is practicable when $\delta =0.003,0.03$. Moreover, it can be concluded that approximation formula (13a) is available if *r* is medium, *m* is medium, $r>\frac{{\left( {m - d} \right) \tau C_{d - 1}^{m - 1} + \left( {\tau - d\tau + d} \right) \sum \limits _{k = m}^{d - 1} {C_{d - 1}^k} }}{{mC_{d - 1}^{m - 1} + \sum \limits _{k = m}^{d - 1} {C_{d - 1}^k} }}$, and $\delta =0.003,0.03$.

**Fig. 4:**
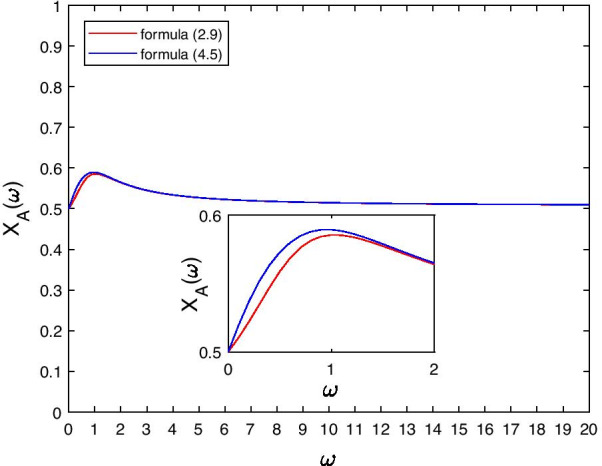
Average abundance function with mutation ${{X}_{A}}(\omega )$ changes with $\omega$ based on preliminary formula (7a) and approximation formula (13a) ($d=15,r=8,c=1,\tau =0.25,\alpha =1,m=8,N=100,\delta =0.03$)

Furthermore, based on the discussions above, we can obtain the curves describing average abundance function with mutation ${{X}_{A}}(\omega )$ based on preliminary formula (7a) and approximation formula (13a) (Fig. 4). In this situation, we set the basic parameters as:$d=15$, $r=8$, $c=1$, $\tau =0.25$, $\alpha =1$, $m=8$, $N=100$, $\delta =0.03$. It should be noted that the curves in Fig. 4 are based on different formulas. The red curve represents ${{X}_{A}}(\omega )$ based on preliminary formula (7a). The blue curve represents ${{X}_{A}}(\omega )$ based on approximate formula (13a). In addition, the insert represents ${{X}_{A}}(\omega )$ when $0 \le \omega \le 2$. On the basis of Fig. 4, two properties can be obtained:

##### **Property 4.1.1**

*The blue curve based on approximation formula (*13a*) is close to the red line based on preliminary formula (*7a*) when *$0 \le \omega \le 2$. *The difference between the two curves is slight but not negligible.*

##### **Property 4.1.2**

*The blue curve based on approximation formula (*13a*) nearly coincides with the red curve based on original formula (*7a*) when* $\omega > 2$. *The difference between the two curves is tiny and can be ignored.*

On the basis of the two properties, it can be concluded that approximation formula (13a) will become more reliable when $\omega$ is larger.

### Strategy *A* is not favoured by selection

#### Large *r* and small *m*

**Fig. 5:**
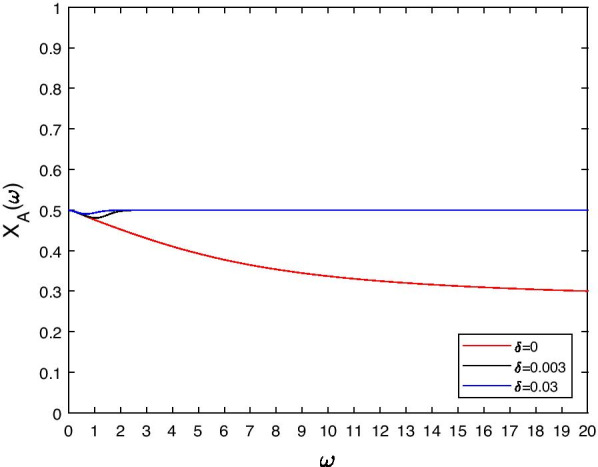
Average abundance function ${{X}_{A}}(\omega )$ changes with selection intensity $\omega$ under different mutations $\delta$ ($d=15,r=10,c=1,\tau =0.25,\alpha =1,m=2,N=100$) ($\delta =0,0.003,0.03$)

On the basis of formula (16) and Fig. 5-1, it can be deduced that there are two conditions to ensure that strategy *A* is not favoured by selection (${{X}_{A}}(\omega )<1/2$). The first condition is large *r* and small *m*. The second condition is medium *r* and medium *m*. Both conditions must satisfy the premise that $r<\frac{{\left( {m - d} \right) \tau C_{d - 1}^{m - 1} + \left( {\tau - d\tau + d} \right) \sum \limits _{k = m}^{d - 1} {C_{d - 1}^k} }}{{mC_{d - 1}^{m - 1} + \sum \limits _{k = m}^{d - 1} {C_{d - 1}^k} }}$.

At this time the first condition is considered. In the beginning, we set the basic parameters as: $d=15$, $r=10$, $c=1$, $\tau =0.25$, $\alpha =1$, $m=2$, $N=100$. It should be noted that $r=10<\frac{{\left( {m - d} \right) \tau C_{d - 1}^{m - 1} + \left( {\tau - d\tau + d} \right) \sum \limits _{k = m}^{d - 1} {C_{d - 1}^k} }}{{mC_{d - 1}^{m - 1} + \sum \limits _{k = m}^{d - 1} {C_{d - 1}^k} }}=11.48$. Then, we can obtain the curves (Fig. 5) describing how average abundance function changes with selection intensity $\omega$ under different mutations $\delta$ ($\delta =0$, $\delta =0.003$ or $\delta =0.03$). On the basis of Fig. 5, two corollaries can be obtained:

##### **Corollary 4.2.1**

*At first, average abundance function decreases rapidly. Then, it remains unchanged basically when* $\delta =0$.

##### **Corollary 4.2.2**

*At first, average abundance function decreases. Then, it increases. Finally, it remains unchanged when *$\delta =0.003$ or $\delta =0.03$.

On the basis of two corollaries, it can be deduced that approximation formula (13a) is practicable when $\delta =0.003,0.03$. Moreover, it can be concluded that approximation formula (13a) is available if *r* is large ,*m* is small, $r<\frac{{\left( {m - d} \right) \tau C_{d - 1}^{m - 1} + \left( {\tau - d\tau + d} \right) \sum \limits _{k = m}^{d - 1} {C_{d - 1}^k} }}{{mC_{d - 1}^{m - 1} + \sum \limits _{k = m}^{d - 1} {C_{d - 1}^k} }}$, and $\delta =0.003,0.03$.

**Fig. 6:**
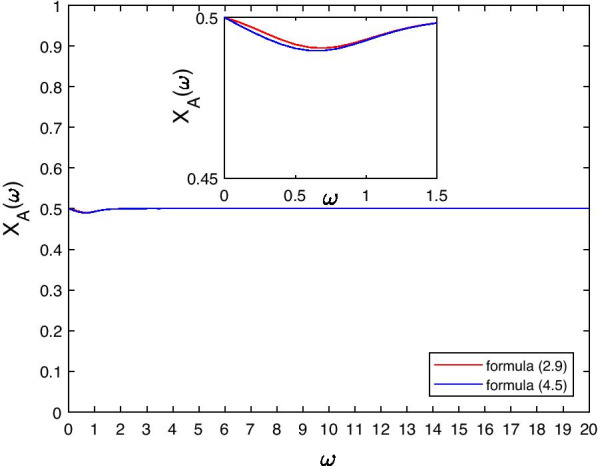
Average abundance function with mutation ${{X}_{A}}(\omega )$ changes with $\omega$ based on preliminary formula (7a) and approximation formula (13a) ($d=15,r=10,c=1,\tau =0.25,\alpha =1,m=2,N=100,\delta =0.03$)

Furthermore, based on the discussions above, we can obtain the curves describing average abundance function ${{X}_{A}}(\omega )$ based on preliminary formula (7a) and approximation formula (13a) (Fig. 6). In this situation, we set the basic parameters as: $d=15$, $r=10$, $c=1$, $\tau =0.25$, $\alpha =1$, $m=2$, $N=100$, $\delta =0.03$. The corresponding analysis is similar to Fig. 4-4. While, it should be noted that the insert in Fig. 5 ,6 represents ${{X}_{A}}(\omega )$ when $0 \le \omega \le 1.5$. On the basis of Fig. 6, two properties can be obtained:

##### **Property 4.2.1**

T*he blue curve based on approximation formula (*13a*) is close to the red line based on preliminary formula (*7a*) when* $0 \le \omega \le 1.5$. *The difference between the two curves is slight but not negligible.*

##### **Property 4.2.2**

*The blue curve based on approximation formula (*13a)* nearly coincides with the red curve based on original formula (*7a*) when* $\omega > 1.5$.* The difference between the two curves is tiny and can be ignored.*

On the basis of the two properties, it can be concluded that approximation formula (13a) will become more reliable when $\omega$ is larger.

#### Medium *r* and medium *m*

**Fig. 7:**
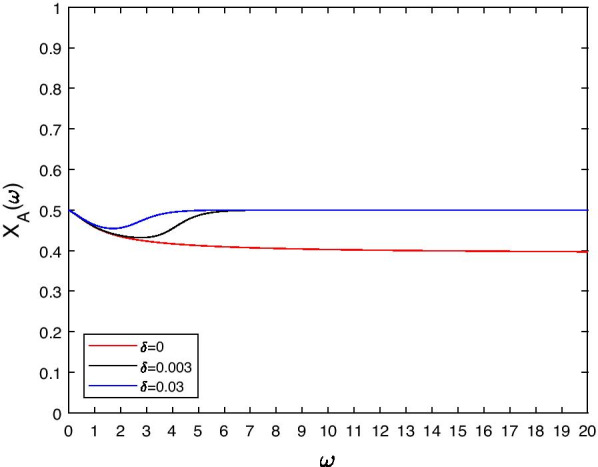
Average abundance function changes with selection intensity $\omega$ under different mutations $\delta$ ($d=15,r=5,c=1,\tau =0.25,\alpha =1,m=5,N=100$) ($\delta =0,0.003,0.03$)

At present the second condition is taken into account. In the beginning , we set the basic parameters as: $d=15$, $r=5$, $c=1$, $\tau =0.25$, $\alpha =1$, $m=5$, $N=100$. It should be noted that $r=5<\frac{{\left( {m - d} \right) \tau C_{d - 1}^{m - 1} + \left( {\tau - d\tau + d} \right) \sum \limits _{k = m}^{d - 1} {C_{d - 1}^k} }}{{mC_{d - 1}^{m - 1} + \sum \limits _{k = m}^{d - 1} {C_{d - 1}^k} }}=8.48$. Then, we can obtain the curves (Fig. 7) describing how average abundance function changes with selection intensity $\omega$ under different mutations $\delta$ ($\delta =0$,$\delta =0.003$ or $\delta =0.03$). On the basis of Figs. 5, 6, 7, two corollaries can be obtained:

##### **Corollary 4.2.3**

*At first, average abundance function decreases rapidly. Then, it remains unchanged basically when* $\delta =0$.

##### **Corollary 4.2.4**

*At first, average abundance function decreases. Then, it increases. Finally, it remains unchanged whe*n $\delta =0.003$ *or* $\delta =0.03$ .

On the basis of two corollaries, it can be deduced that approximation formula (13a) is practicable when $\delta =0.003,0.03$. Moreover, it can be concluded that approximation formula (13a) is available if *r* is medium, *m* is medium, $r<\frac{d\sum \limits _{k=m}^{d-1}{C_{d-1}^{k}}}{mC_{d-1}^{m-1}+\sum \limits _{k=m}^{d-1}{C_{d-1}^{k}}}$, and $\delta =0.003,0.03$.

**Fig. 8:**
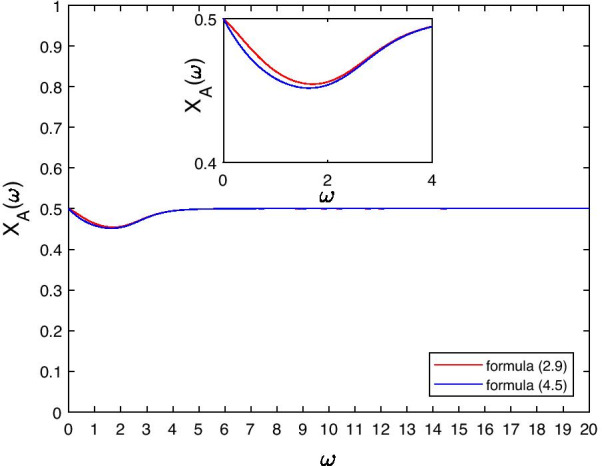
Average abundance function with mutation ${{X}_{A}}(\omega )$ changes with $\omega$ based on original formula (7a) and approximation formula (13a) ($d=15,r=5,c=1,\tau =0.25,\alpha =1,m=5,N=100,\delta =0.03$)

Furthermore, based on the discussions above, we can obtain the curves describing average abundance function ${{X}_{A}}(\omega )$ based on preliminary formula (7a) and approximation formula (13a) (Fig. 8). In this situation, we set the basic parameters as: $d=15$, $r=5$, $c=1$, $\tau =0.25$, $\alpha =1$, $m=5$, $N=100$, $\delta =0.03$. The corresponding analysis is similar to Fig. 6. While, it should be noted that the insert in Fig. 5-8 represents ${{X}_{A}}(\omega )$ when $0 \le \omega \le 4$. On the basis of Fig. 8, two properties can be obtained:

##### **Property 4.2.3**

*The blue curve based on approximation formula (*13a*) is close to the red line based on preliminary formula (*7a*) when* $0 \le \omega \le 4$. *The difference between the two curves is slight but not negligible.*

##### **Property 4.2.4**

*The blue curve based on approximation formula (*13a*) nearly coincides with the red curve based on original formula (*7a*) when* $\omega > 4$. *The difference between the two curves is tiny and can be ignored.*

On the basis of the two properties, it can be concluded that approximation formula (13a) will become more reliable when $\omega$ is larger.

### Brief Summary

Combined with the above discussions, three inferences can be obtained:

#### Inference 4.1

Approximation formula (13a) is not available when *r* is small, *m* is large, and $\delta =0.03$. Approximation formula (13a) is available in other cases.

#### Inference 4.2

Approximation formula (13a) will become more reliable when $\omega$ is larger.

#### Inference 4.3

The feasibility of approximate formula (13a) is the highest when *r* is large and *m* is small.

Then we carry on the research on Inference 4.3. It can be deduced from the above discussions that approximation formula (13a) will be reliable if $\omega >2$ when *r* is medium, *m* is medium, and $r>\frac{{\left( {m - d} \right) \tau C_{d - 1}^{m - 1} + \left( {\tau - d\tau + d} \right) \sum \limits _{k = m}^{d - 1} {C_{d - 1}^k} }}{{mC_{d - 1}^{m - 1} + \sum \limits _{k = m}^{d - 1} {C_{d - 1}^k} }}$. In addition, it will be reliable if $\omega >1.5$ when *r* is large, *m* is small, and $r<\frac{{\left( {m - d} \right) \tau C_{d - 1}^{m - 1} + \left( {\tau - d\tau + d} \right) \sum \limits _{k = m}^{d - 1} {C_{d - 1}^k} }}{{mC_{d - 1}^{m - 1} + \sum \limits _{k = m}^{d - 1} {C_{d - 1}^k} }}$. Moreover, it will be reliable if $\omega >4$ when *r* is medium, *m* is medium, and $r<\frac{{\left( {m - d} \right) \tau C_{d - 1}^{m - 1} + \left( {\tau - d\tau + d} \right) \sum \limits _{k = m}^{d - 1} {C_{d - 1}^k} }}{{mC_{d - 1}^{m - 1} + \sum \limits _{k = m}^{d - 1} {C_{d - 1}^k} }}$.

On the whole, it can be concluded that the practicability of approximate formula (13a) is most remarkable when *r* is large and *m* is small. The reason lies in the fact only $\omega >1.5$ is needed in this condition while $\omega >2$ or $\omega >4$ is needed in other cases.

Moreover, the inference 4.3 can be explained based on expected payoff function $\pi \left( \centerdot \right)$ defined by formula (10a). On the basis of formula (10a) , it can be deduced that both ${{\pi }_{A}}(i+1)$ and ${{\pi }_{B}}(i)$ will increase with *r* and decrease with *m*. Thus it can be concluded that $\pi \left( \centerdot \right)$ under the condition of large *r* and small *m* will be larger than those under the other two conditions.

In addition, based on the discussions in section 4, it can be deduced that the most important premise to ensure the establishment of approximation formula (13a) is condition (11) and (12):

$$\begin{aligned} {e^{ - \omega (\alpha - {\pi _A}(i + 1))}}\gg & {} 1, {e^{ - \omega (\alpha - {\pi _B}(i))}} \gg 1 \\ \delta {e^{ - \omega (\alpha - {\pi _A}(i + 1))}}> & {} 1,\delta {e^{ - \omega (\alpha - {\pi _B}(i))}} > 1 \end{aligned}$$

On the basis of (11) and (12), it can be deduced that:

16a $$\begin{aligned} \omega \left[ \pi \left( \centerdot \right) -\alpha \right]&\gg 0 \end{aligned}$$

16b $$\begin{aligned} \omega&>\frac{\ln \left( \frac{1}{\delta } \right) }{\pi \left( \centerdot \right) -\alpha } \end{aligned}$$

Then, it can be deduced that even small $\omega$ can guarantee the establishment of formulas (16a) and (16b) if $\pi \left( \centerdot \right)$ is large enough. Furthermore, it can be obtained that even small $\omega$ can guarantee the approximate formula (13a) if $\pi \left( \centerdot \right)$ is large enough. Combined with the above discussions, inference 4.3 can be explained.

Moreover, it has been deduced that the approximation expression of average abundance function when $\omega \rightarrow 0$ is ${{X}_{A}}(\omega )=\frac{1}{2}+\omega \frac{1-\delta }{1+\delta } \frac{1}{{{2}^{d+2}}}\sum \limits _{k=0}^{d-1}{C_{d-1}^{k}}\left( {{a}_{k}}-{{b}_{k}} \right)$ when selection intensity $\omega$ is sufficient small($\omega \rightarrow 0$). On the whole, by combing this conclusion with proposition 4-1, it can be concluded that:

#### **Proposition 5-1**

*Approximate expression of average abundance function* ${{X}_{A}}(\omega )$ *can be defined as follows:*

   

16c $$X_{A} (\omega ) \approx {\text{ }}\left\{ {\begin{array}{*{20}c} {\frac{1}{2} + \omega \frac{{1 - \delta }}{{1 + \delta }}\frac{1}{{2^{{d + 2}} }}\sum\limits_{{k = 0}}^{{d - 1}} {C_{{d - 1}}^{k} } \left( {a_{k} - b_{k} } \right)} & {when\begin{array}{*{20}l} {} \hfill \\ \end{array} \omega \begin{array}{*{20}l} {} \hfill \\ \end{array} is\begin{array}{*{20}l} {} \hfill \\ \end{array} small\begin{array}{*{20}l} {} \hfill \\ \end{array} enough} \\ {\frac{1}{N}\frac{{\sum\limits_{{j = 1}}^{N} {j\mathop {\mathop \Pi \limits^{{j - 1}} }\limits_{{i = 0}} {\mkern 1mu} h_{a} (i,\omega )} }}{{1 + \sum\limits_{{j = 1}}^{N} {\mathop {\mathop \Pi \limits^{{j - 1}} }\limits_{{i = 0}} {\mkern 1mu} h_{a} (i,\omega )} }}} & {when\begin{array}{*{20}l} {} \hfill \\ \end{array} \omega \begin{array}{*{20}l} {} \hfill \\ \end{array} is\begin{array}{*{20}l} {} \hfill \\ \end{array} large\begin{array}{*{20}l} {} \hfill \\ \end{array} enough} \\ \end{array} } \right.$$

16d $$\begin{aligned} {h_a}\left( {i,\omega } \right)= & {} \frac{{N - i}}{{i + 1}} \times \frac{{1 + \delta {e^{ - \omega \left( {\alpha - {\pi _B}\left( i \right) } \right) }}}}{{1 + \delta {e^{ - \omega \left( {\alpha - {\pi _A}\left( {i + 1} \right) } \right) }}}} \times {e^{ - \omega \left( {{\pi _B}\left( i \right) - {\pi _A}\left( {i + 1} \right) } \right) }} \end{aligned}$$

On the basis of proposition 5-1, it can be obtained that we have obtained the approximate expression of average abundance function ${{X}_{A}}(\omega )$ under different levels of selection intensity $\omega$.

## Numerical simulation on average abundance function

It can be seen from the above discussions that different parameters will have different effects on average abundance function. Furthermore, this is a problem worthy of discussion. By numerical simulation, we can explore the influence of parameters on average abundance function based on multi-player threshold public goods evolutionary game model under redistribution mechanism.

At the beginning, we will consider the specific value of parameters. The basic parameters are set as: $d=15$, $r=8$, $c=1$, $\tau =0.25$, $\alpha =1$, $\delta =0.03$, $N=100$. It should be noted that the most important parameter in threshold public goods model is the threshold *m*. On one hand, the threshold *m* plays an important role in determining whether strategy *A* is favoured by selection (${{X}_{A}}(\omega )>1/2$) or not (${{X}_{A}}(\omega )<1/2$). On the other hand, the threshold *m* affect the influence of *d*,*r*,*c*,$\alpha$ on average abundance function ${{X}_{A}}(\omega )$. So the influence of threshold *m* should be taken into consideration carefully.

**Fig. 9:**
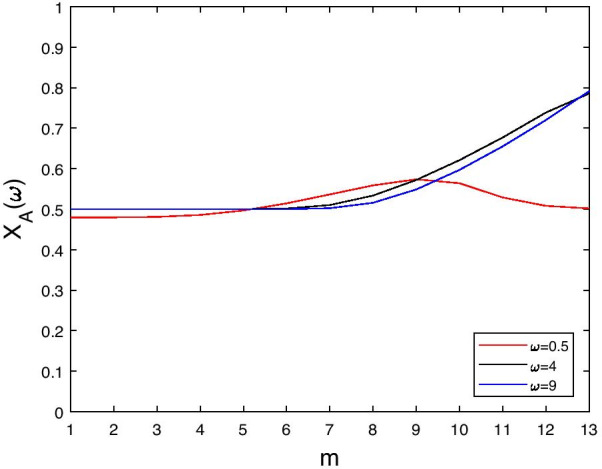
Average abundance function ${{X}_{A}}(\omega )$ changes with *m* ($d=15$,$r=8$,$c=1$,$\tau =0.25$,$\alpha =1$,$N=100$)

We will draw curves describing how average abundance function changes with threshold *m* (see Fig. 9). Then based on the analysis of Fig. 9, two properties can be obtained:

### **Property 5.1**

*When* $\omega =0.5$, *average abundance function will increase with m if* $m\le {\text {int}}\left\{ \frac{d}{4\left( r-\tau \right) }\left[ r-2\tau -1+\sqrt{{{\left( r-2\tau +3 \right) }^{2}}+8\left( \tau -1 \right) } \right] \right\} +1$ *and it will decrease with m if* $m> {\text {int}}\left\{ \frac{d}{4\left( r-\tau \right) }\left[ r-2\tau -1+\sqrt{{{\left( r-2\tau +3 \right) }^{2}}+8\left( \tau -1 \right) } \right] \right\} +1$. *Furthermore, average abundance function will reach the maximum value when* $m={\text {int}}\left\{ \frac{d}{4\left( r-\tau \right) }\left[ r-2\tau -1+\sqrt{{{\left( r-2\tau +3 \right) }^{2}}+8\left( \tau -1 \right) } \right] \right\} +1$.

### **Property 5.2**

*When* $\omega =4$ *and* $\omega =9$, *average abundance function will increase with m*.

The deduction for property 5.1 is a little long and it is placed in Appendix A. Furthermore, based on for property 5.1, we will consider the characteristics of average abundance function when two special value of *m* are set: $m=1$ and $m=14$. We have already deduced that the approximate expression of ${{X}_{A}}(\omega )$ when $\omega$ is small can be defined as follows:

17 $$\begin{aligned} {{X}_{A}}(\omega )= & {} \frac{1}{2}+\omega \frac{{1 - \delta }}{{1 + \delta }} \frac{1}{{{2}^{d+2}}}\frac{c}{d}\left[ \left( \frac{m\left( r-\tau \right) +d\tau }{r-\tau +d\tau }C_{d-1}^{m-1}+\sum \limits _{k=m}^{d-1}{C_{d-1}^{k}} \right) \left( r-\tau +d\tau \right) \right. \nonumber \\&\left. -d\sum \limits _{k=m}^{d-1}{C_{d-1}^{k}} \right] \end{aligned}$$

On one hand, when $m=1$ ,the formula (17) can be simplified as follows:

18 $$\begin{aligned} {X_A}\left( \omega \right)= & {} \frac{1}{2} +\omega \frac{{1 - \delta }}{{1 + \delta }} \frac{1}{{{2^{d + 2}}}}\frac{c}{d}\left[ \left( {\frac{{\left( {r - \tau } \right) + d\tau }}{{r - \tau + d\tau }}C_{d - 1}^0 + \sum \limits _{k = 1}^{d - 1} {C_{d - 1}^k} } \right) \left( {r - \tau + d\tau } \right) \right. \nonumber \\&\left. - d\sum \limits _{k = 1}^{d - 1} {C_{d - 1}^k} \right] \nonumber \\= & {} \frac{1}{2} + \omega \frac{{1 - \delta }}{{1 + \delta }} \frac{1}{{{2^{d + 2}}}}\frac{c}{d}\left[ {\left( {1 + {2^{d - 1}} - 1} \right) \left( {r - \tau + d\tau } \right) - d\left( {{2^{d - 1}} - 1} \right) } \right] \nonumber \\= & {} \frac{1}{2} + \omega \frac{{1 - \delta }}{{1 + \delta }} \frac{1}{{{2^{d + 2}}}}\frac{c}{d}\left[ {{2^{d - 1}}\left( {r - \tau + d\tau } \right) - d\left( {{2^{d - 1}} - 1} \right) } \right] \nonumber \\\approx & {} \frac{1}{2} + \omega \frac{{1 - \delta }}{{1 + \delta }} \frac{1}{{{2^{d + 2}}}}\frac{c}{d}\left[ {{2^{d - 1}}\left( {r - \tau + d\tau - d} \right) } \right] \nonumber \\= & {} \frac{1}{2} + \omega \frac{{1 - \delta }}{{1 + \delta }} \frac{1}{8}\frac{c}{d}\left( {r - \tau + d\tau - d} \right) \end{aligned}$$

By inserting the values of parameters into formula (18), it can be deduced that:

19 $$\begin{aligned} {{X}_{A}}(\omega )\approx 0.4863 \end{aligned}$$

Then we can obtain that ${{X}_{A}}(\omega )\approx 0.4863$ when $m=1$.

On the other hand, when $m=14$, the formula (17) can be simplified as follows:

20 $$\begin{aligned} {X_A}\left( \omega \right)= & {} \frac{1}{2} + \omega \frac{{1 - \delta }}{{1 + \delta }} \frac{1}{{{2^{d + 2}}}}\frac{c}{d}\left[ \left( {\frac{{14\left( {r - \tau } \right) + d\tau }}{{r - \tau + d\tau }}C_{14}^{13} + \sum \limits _{k = 14}^{14} {C_{14}^k} } \right) \left( {r - \tau + d\tau } \right) \right. \nonumber \\&\left. - d\sum \limits _{k = 14}^{14} {C_{14}^k} \right] \nonumber \\= & {} \frac{1}{2} + \omega \frac{{1 - \delta }}{{1 + \delta }} \frac{1}{{{2^{d + 2}}}}\frac{c}{d}\left[ {\left( {14 \times \frac{{14\left( {r - \tau } \right) + d\tau }}{{r - \tau + d\tau }} + 1} \right) \left( {r - \tau + d\tau } \right) - d} \right] \nonumber \\= & {} \frac{1}{2} + \omega \frac{{1 - \delta }}{{1 + \delta }} c\left[ {\left( {14 \times \frac{{14\left( {r - \tau } \right) + d\tau }}{{r - \tau + d\tau }} + 1} \right) \frac{{r - \tau + d\tau }}{{{2^{d + 2}}d}} - \frac{1}{{{2^{d + 2}}}}} \right] \end{aligned}$$

It can be deduced that the second item on the right side of formula (20) will $\rightarrow 0$ because $\frac{r-\tau +d\tau }{{{2}^{d+2}}}\rightarrow 0$ and $\frac{1}{{{2}^{d+2}}}\rightarrow 0$. So there will be ${{X}_{A}}(\omega )\approx 0.5$ when $m=14$.

Furthermore, it can be concluded that ${{X}_{A}}(\omega )\approx 0.4863$ when $m=1$ and ${{X}_{A}}(\omega )\approx 0.5$ when $m=14$. It should be noted that this conclusion is very close to the corresponding values in Fig. 9.

Moreover, we can obtain $m=9$ by inserting the basic parameters into formula $m={\text {int}}\left\{ \frac{d}{4\left( r-\tau \right) }\left[ r-2\tau -1+\sqrt{{{\left( r-2\tau +3 \right) }^{2}}+8\left( \tau -1 \right) } \right] \right\} +1$ . Then, it can be deduced that when $\omega =0.5$, average abundance function will increase with *m* if $m\le 9$ and it will decrease with *m* if $m>9$. Furthermore, average abundance function will reach the maximum value when $m=9$. These conclusions are consistent with the information revealed in Fig. 9. Combining the above discussions , it can be concluded that property 5.1 is reliable.

Based on the intrinsic characteristics of threshold *m*, the property 5.2 can be explained. The increase of threshold *m* means that there must be more collaborators in the group to ensure that threshold public goods game will go on. So average abundance function ${{X}_{A}}(\omega )$ will increase with *m*.

Based on the discussions above, we set $m=4$ and $m=10$. On one hand, the $m=4$ corresponds to the situation that average abundance function is in an upward trend when $\omega =0.5$. The $m=10$ corresponds to the situation that average abundance function is on a downward trend when $\omega =0.5$. On the other hand, the $m=4$ represents that strategy *A* is not favoured by selection (${{X}_{A}}(\omega )<1/2$). The $m=10$ represents that strategy *A* is favoured by selection(${{X}_{A}}(\omega )>1/2$).

Furthermore, the numerical simulation will be carried out after the value of threshold *m* is selected. In order to study the trend of average abundance function under different levels of selection intensity $\omega$, we select $\omega =0.5$, $\omega =4$ and $\omega =9$ . In addition, it should be noted that we will analyze the trend of average abundance function when *d*, *r*, *c*, $\alpha$ changes respectively. It means that when we analyze the effect of a particular parameter (such as *d*), the other parameters (*r*, *c*, $\alpha$) will remain unchanged. Other analyses are similar. Moreover, it should be noted that both the situation when $m=4$ and the situation when $m=10$ will be taken into consideration. Then, we can obtain the curves describing how average abundance function changes with parameters.

### The influence of *d* on average abundance function

**Fig. 10:**
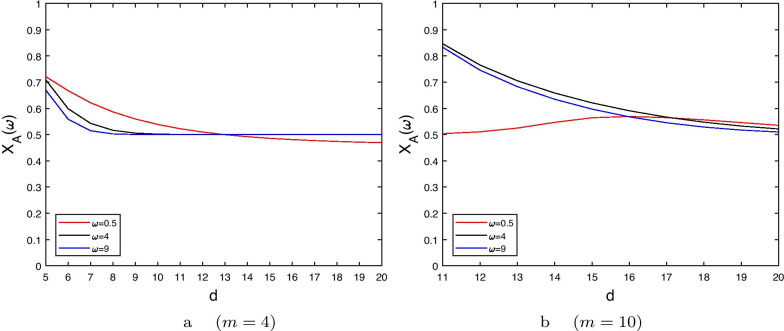
Average abundance function ${{X}_{A}}(\omega )$ changes with *d* ($r=8,c=1,\tau =0.25,\alpha =1,N=100$)

It should be noted that $1<m<d$ in multi-player threshold public goods evolutionary game model. Furthermore it can be concluded that $d \ge 5$ when $m=4$ and $d \ge 11$ when $m=10$. Then, we can obtain Fig. 10 describing how average abundance function changes with *d*. Moreover, four corollaries can be obtained from the analysis of Fig. 10.

#### **Corollary 5.1.1**

*If* $m=4$*, average abundance function will decrease at first and then remain stable at 1/2 with the increase of* *d* *when* $\omega =0.5$.

#### **Corollary 5.1.2**

*If* $m=10$*, average abundance function will increase at first and then decrease with the increase of* *d when* $\omega =0.5$.

#### **Corollary 5.1.3**

*If* $m=4$*, average abundance function will decrease at first and then remain stable at 1/2 with the increase of* *d* *when* $\omega =4$ *and* $\omega =9$.

#### **Corollary 5.1.4**

*If* $m=10$*, average abundance function will decrease at first and then get close to 1/2 with the increase of* *d* *when* $\omega =4$ *and* $\omega =9$.

#### **Corollary 5.1.5**

*The average abundance function when* $m=10$ *is larger than the average abundance function when* $m=4$ *if* $\omega =4$ *or* $\omega =9$.* In other words,* ${X_A}(\omega )\left| {_{m=10}} \right. > {X_A}(\omega )\left| {_{m = 4}} \right.$ *if *$\omega =4$ *or* $\omega =9$.

#### The explanation for Corollary 5.1.1

The corollary 5.1.1 when $m=4$ can be explained by simplifying the formula (17) as:

21 $$\begin{aligned} {X_A}\left( \omega \right)= & {} \frac{1}{2} + \omega \frac{{1 - \delta }}{{1 + \delta }} \frac{1}{{{2^{d + 2}}}}\frac{c}{d}\left[ \left( {m\left( {r - \tau } \right) + d\tau } \right) C_{d - 1}^{m - 1} \right. \nonumber \\&\left. + \left( {r - \tau + d\tau - d} \right) \sum \limits _{k = m}^{d - 1} {C_{d - 1}^k} \right] \end{aligned}$$

Based on formula (21), it is easy to deduce that average abundance function will decrease with *d*:

22 $$\begin{aligned} &{X_A}\left( \omega \right) \left| {_{d + 1}} \right. - {X_A}\left( \omega \right) \left| {_d} \right. \nonumber \\&\quad = \omega \frac{{1 - \delta }}{{1 + \delta }} \frac{1}{{{2^{d + 2}}}}\left\{ \frac{c}{{d + 1}}\left[ \left( {4\left( {r - \tau } \right) + d\tau + \tau } \right) C_d^3\right. \right. \nonumber \\&\qquad \left. \left. + \left( {r - \tau + d\tau + \tau - d - 1} \right) \sum \limits _{k = 4}^{d - 1} {C_d^k} \right] \right\} \nonumber \\&\qquad - \omega \frac{{1 - \delta }}{{1 + \delta }} \frac{1}{{{2^{d + 2}}}}\left\{ \frac{c}{d}\left[ \left( {4\left( {r - \tau } \right) + d\tau } \right) C_{d - 1}^3 \right. \right. \nonumber \\&\qquad \left. \left. + \left( {r - \tau + d\tau - d} \right) \sum \limits _{k = 4}^{d - 1} {C_{d - 1}^k} \right] \right\} \nonumber \\&\quad < 0 \end{aligned}$$

Based on formula (22), it can be deduced that average abundance function will decrease with the increase of *d*.

Moreover, a lot of individuals choosing strategy *B* will turn to choose strategy *A* because of mutation when *d* is large enough($d>14$). Furthermore, the combination of these two conditions leads to the fact that the proportion of individuals choosing strategy *A* and the proportion of individuals choosing strategy *B* reach a equilibrium. Therefore average abundance function remains stable at 1/2 when *d* increases to a very high level($d>14$).

#### The explanation for Corollary 5.1.2

On one hand, based on formula (21), it can be deduced that when $d<14$, the average abundance function will increase with *d*:

23a $$\begin{aligned}&{X_A}\left( \omega \right) \left| {_{d + 1}} \right. - {X_A}\left( \omega \right) \left| {_d} \right. \nonumber \\&\quad = \omega \frac{{1 - \delta }}{{1 + \delta }} \frac{1}{{{2^{d + 2}}}}\left\{ \frac{c}{{d + 1}}\left[ \left( {10\left( {r - \tau } \right) + d\tau + \tau } \right) C_d^9 \right. \right. \nonumber \\&\qquad \left. \left. + \left( {r - \tau + d\tau + \tau - d - 1} \right) \sum \limits _{k = 10}^{d - 1} {C_d^k} \right] \right\} \nonumber \\&\qquad - \omega \frac{{1 - \delta }}{{1 + \delta }} \frac{1}{{{2^{d + 2}}}}\left\{ \frac{c}{d}\left[ \left( {10\left( {r - \tau } \right) + d\tau } \right) C_{d - 1}^9 \right. \right. \nonumber \\&\qquad \left. \left. + \left( {r - \tau + d\tau - d} \right) \sum \limits _{k = 10}^{d - 1} {C_{d - 1}^k} \right] \right\} \nonumber \\&\quad > 0 \end{aligned}$$

On the other hand, based on formula (21), it can be deduced that when $d>15$, the average abundance function will decrease with *d*:

23b $$\begin{aligned}&{X_A}\left( \omega \right) \left| {_{d + 1}} \right. - {X_A}\left( \omega \right) \left| {_d} \right. \nonumber \\&\quad = \omega \frac{{1 - \delta }}{{1 + \delta }} \frac{1}{{{2^{d + 2}}}}\left\{ \frac{c}{{d + 1}}\left[ \left( {10\left( {r - \tau } \right) + d\tau + \tau } \right) C_d^9 \right. \right. \nonumber \\&\qquad \left. \left. + \left( {r - \tau + d\tau + \tau - d - 1} \right) \sum \limits _{k = 10}^{d - 1} {C_d^k} \right] \right\} \nonumber \\&\qquad - \omega \frac{{1 - \delta }}{{1 + \delta }} \frac{1}{{{2^{d + 2}}}}\left\{ \frac{c}{d}\left[ \left( {10\left( {r - \tau } \right) + d\tau } \right) C_{d - 1}^9 \right. \right. \nonumber \\&\qquad \left. \left. + \left( {r - \tau + d\tau - d} \right) \sum \limits _{k = 10}^{d - 1} {C_{d - 1}^k} \right] \right\} \nonumber \\&\quad < 0 \end{aligned}$$

Based on formula (23a) and (23b), it can be deduced that average abundance function will increase at first and then decrease with the increase of *d*.

#### The explanation for Corollary 5.1.3

The main point of analysis lies in the fact that individuals will become more rational when selection intensity $\omega$ is large($\omega =4$ and $\omega =9$). In other words, more individuals in the group will choose strategy *B* when selection intensity $\omega$ becomes larger. Then, it can be deduced that the proportion of individuals choosing strategy *A* in the group will decrease with the increase of *d*. Therefore, average abundance function will decrease.

Moreover, a lot of individuals choosing strategy *B* will turn to choose strategy *A* because of mutation when *d* is large enough($d>8$). Furthermore, the combination of these two conditions leads to the fact that the proportion of individuals choosing strategy *A* and the proportion of individuals choosing strategy *B* reach a equilibrium. Therefore average abundance function remains stable at 1/2 when *d* increases to a very high level($d>8$).

#### The explanation for Corollary 5.1.4

The reason for corollary 5.1.4 is similar to the reason for corollary 5.1.3. However, it should be noted that average abundance function will get close to 1/2 (not remain unchanged at 1/2) when *d* increases to a very high level. Furthermore, getting close to 1/2 is different from remaining unchanged at 1/2. This difference is closely related to the intrinsic characteristics of threshold. When *m* increases, there must be more collaborators in the group to ensure that threshold public goods game can continue. Therefore, average abundance function when $m=10$ is larger than average abundance function when $m=4$ when $\omega =4$ and $\omega =9$. On the whole, it can be deduced that the increase of threshold *m* will slow down the decreasing of average abundance function. Then it can be deduced that the rate of decline when $m=10$ is lower than the rate of decline when $m=4$. Then average abundance function will get close to 1/2 rather than remain unchanged at 1/2 when $m=10$.

#### The explanation for Corollary 5.1.5

Based on the intrinsic characteristics of threshold, corollary 5.1.5 can be explained. The increase of threshold means that there must be more collaborators in the group to ensure that threshold public goods game will go on. So it can be deduced that the increase of threshold *m* ($m=4$ increases to $m=10$) will inhibit the downward trend of average abundance function. In other words, the decreasing rate of average abundance function when $m=4$ will be faster than that when $m=10$. So there will be ${X_A}(\omega )\left| {_{m=10}} \right. > {X_A}(\omega )\left| {_{m = 4}} \right.$.

### The influence of *r* on average abundance function

**Fig. 11:**
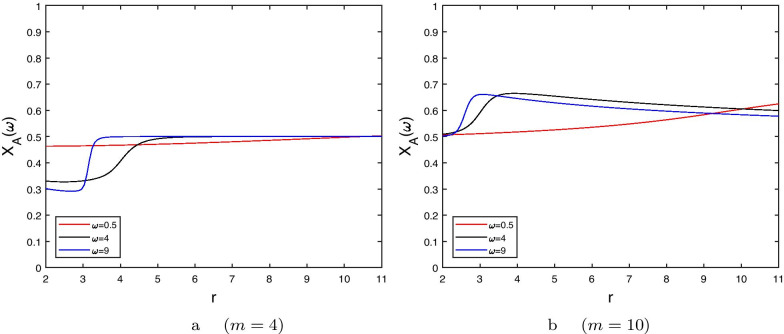
Average abundance function ${{X}_{A}}(\omega )$ changes with *r* ($d=15,c=1,\tau =0.25,\alpha =1,N=100$)

We can obtain Fig. 11 describing how average abundance function changes with *r*. Moreover, three corollaries can be obtained from the analysis of Fig. 11.

#### **Corollary 5.2.1**

*If* $m=4$*, average abundance function will increase with* *r* *when* $\omega =0.5$.* The trend for the situation when* $m=10$ *is similar to this. However, the trend for the former is close to linear change while the trend for the latter is not.*

#### **Corollary 5.2.2**

*If* $m=4$, *average abundance function will decrease at first, and then increase, at last remain unchanged at 1/2 with the increase of r* when $\omega =4$ *or* $\omega =9$.

#### **Corollary 5.2.3**

*If* $m=10$, *average abundance function will increase at first, and then decrease with the increase of* *r* when $\omega =4$ *or* $\omega =9$.

#### The explanation for Corollary 5.2.1

The corollary 5.2.1 when $\omega =0.5$ and $m=4$ can be explained based on formula (17):

$$\begin{aligned} {X_A}\left( \omega \right)= & {} \frac{1}{2} + \omega \frac{{1 - \delta }}{{1 + \delta }} \frac{1}{{{2^{d + 2}}}}\frac{c}{d}\left[ \left( {\frac{{m\left( {r - \tau } \right) + d\tau }}{{r - \tau + d\tau }}C_{d - 1}^{m - 1} + \sum \limits _{k = m}^{d - 1} {C_{d - 1}^k} } \right) \left( {r - \tau + d\tau } \right) \right. \\&\left. - d\sum \limits _{k = m}^{d - 1} {C_{d - 1}^k} \right] \end{aligned}$$

Based on formula (17), it can be deduced that:

24 $$\begin{aligned} \frac{{\partial {X_A}\left( \omega \right) }}{{\partial r}} = \omega \frac{{1 - \delta }}{{1 + \delta }} \frac{1}{{{2^{d + 2}}}}\frac{c}{d}\left[ {mC_{d - 1}^{m - 1} + \sum \limits _{k = m}^{d - 1} {C_{d - 1}^k} } \right] \end{aligned}$$

So average abundance function will increase with *r* when $\omega =0.5$. In addition, the trend is close to linear change.

The reason why average abundance function will increase with *r* when $\omega =0.5$ if $m=10$ is similar to the situation when $m=4$. But it should be noted the corresponding trend is not linear change. Furthermore, it can be deduced that the applicability of formula (17) is limited when $m=10$. It indicates that the complete applicability of formula (17) requires that *m* should be smaller (at lest $m<10$).

#### The explanation for Corollary 5.2.2

First of all, we will try to explain why average abundance function will decrease with *r* when $r<3$. Based on the characteristics of ${{\pi }_{A}}(i+1)$ and ${{\pi }_{B}}(i)$, it can be deduced that the inequality ${{\pi }_{B}}(i)>{{\pi }_{A}}(i+1)$ holds in most cases when $r<3$. This means that in the process of the increase of *r*, the ${{\pi }_{B}}(i)$ will become larger than aspiration level $\alpha$ soon, while the ${{\pi }_{A}}(i+1)$ is still smaller than aspiration level $\alpha$ at the same time. In other words, ${{\pi }_{B}}(i)>\alpha$ and ${{\pi }_{A}}(i+1)<\alpha$. Therefore, more individuals will choose strategy *B*. So average abundance function will decrease with *r* when $r<3$.

Then, we will try to explain why average abundance function will increase with *r* when $r>3$. Based on the expected payoff function $\pi \left( \centerdot \right)$ defined by formula (10a) and the function $h\left( i,\omega \right)$ defined by formula (7a), this phenomenon can be explained.

Based on formula (10a), we can obtain the derivative of ${{\pi }_{A}}(i+1)$ and ${{\pi }_{B}}(i)$ with respect to *r*. In addition, we can compare the derivative of ${{\pi }_{A}}(i+1)$ with the derivative of ${{\pi }_{B}}(i)$ :

24a $$\begin{aligned} \frac{{\partial {\pi _A}\left( {i + 1} \right) }}{{\partial r}}= & {} \frac{{\partial \left[ {\sum \limits _{k = m - 1}^{d - 1} {\frac{{C_i^kC_{N - i - 1}^{d - 1 - k}}}{{C_{N - 1}^{d - 1}}}} \left( {\frac{{k + 1}}{d}\left( {r - \tau } \right) c + \tau c} \right) } \right] }}{{\partial r}}\nonumber \\= & {} \sum \limits _{k = m - 1}^{d - 1} {\frac{{C_i^kC_{N - i - 1}^{d - 1 - k}}}{{C_{N - 1}^{d - 1}}}} \frac{{k + 1}}{d}c \end{aligned}$$

24b $$\begin{aligned} \frac{{\partial {\pi _B}\left( i \right) }}{{\partial r}}= & {} \frac{{\partial \left[ {\sum \limits _{k = m}^{d - 1} {\frac{{C_i^kC_{N - i - 1}^{d - 1 - k}}}{{C_{N - 1}^{d - 1}}}} \left( {\frac{k}{d}\left( {r - \tau } \right) c + c} \right) } \right] }}{{\partial r}}\nonumber \\= & {} \sum \limits _{k = m}^{d - 1} {\frac{{C_i^kC_{N - i - 1}^{d - 1 - k}}}{{C_{N - 1}^{d - 1}}}} \frac{k}{d}c \end{aligned}$$

24c $$\begin{aligned} \frac{{\partial {\pi _A}\left( {i + 1} \right) }}{{\partial r}}= & {} \sum \limits _{k = m - 1}^{d - 1} {\frac{{C_i^kC_{N - i - 1}^{d - 1 - k}}}{{C_{N - 1}^{d - 1}}}} \frac{{k + 1}}{d}c\nonumber \\> & {} \sum \limits _{k = m}^{d - 1} {\frac{{C_i^kC_{N - i - 1}^{d - 1 - k}}}{{C_{N - 1}^{d - 1}}}} \frac{{k + 1}}{d}c\nonumber \\> & {} \sum \limits _{k = m}^{d - 1} {\frac{{C_i^kC_{N - i - 1}^{d - 1 - k}}}{{C_{N - 1}^{d - 1}}}} \frac{k}{d}c\nonumber \\= & {} \frac{{\partial {\pi _B}\left( i \right) }}{{\partial r}} \end{aligned}$$

Based on formula (24a), it can be concluded that both ${{\pi }_{A}}(i+1)$ and ${{\pi }_{B}}(i)$ will increase with *r*. In addition, the increasing rate of ${{\pi }_{A}}(i+1)$ will be higher than that of ${{\pi }_{B}}(i)$.

Based on the discussions above, it can be deduced that both ${{\pi }_{A}}(i+1)$ and ${{\pi }_{B}}(i)$ will become larger than aspiration level $\alpha$ when *r* increases to a very high level ($r>3$). In other words, ${{\pi }_{A}}(i+1)>\alpha$, ${{\pi }_{B}}(i)>\alpha$. In addition, the increasing rate of ${{\pi }_{A}}(i+1)$ with respect to *r* is larger than that of ${{\pi }_{B}}(i)$. So average abundance function will increase with *r* when $r>3$.

Furthermore, we will consider the situation when $r>4$. On the basis of formula (10a),it can be deduced that both ${{\pi }_{A}}(i+1)$ and ${{\pi }_{B}}(i)$ will increase to very high level when $r>4$. Moreover, both $\delta {{e}^{-\omega (\alpha -\pi \left( \centerdot \right) )}}$ and ${{e}^{-\omega (\alpha -\pi \left( \centerdot \right) )}}$ will increase to very high level when $r>4$. It can be seen from the above discussions that the following inequalities will be established:

24d $$\begin{aligned} {{e}^{-\omega (\alpha -\pi \left( \centerdot \right) )}}&>>1 \end{aligned}$$

24e $$\begin{aligned} \delta {{e}^{-\omega (\alpha -\pi \left( \centerdot \right) )}}&>>1 \end{aligned}$$

Then, on the basis of formula (24d), function $h\left( i,\omega \right)$ will gradually degenerate to the following expression:

25 $$\begin{aligned} &\left\{ \begin{aligned}&h(i,\omega )=\frac{N-i}{i+1}\times \frac{1+{{e}^{-\omega (\alpha -{{\pi }_{A}}(i+1))}}}{1+{{e}^{-\omega (\alpha -{{\pi }_{B}}(i))}}}\times \frac{1+\delta {{e}^{-\omega (\alpha -{{\pi }_{B}}(i))}}}{1+\delta {{e}^{-\omega (\alpha -{{\pi }_{A}}(i+1))}}} \\&{{e}^{-\omega (\alpha -\pi (\centerdot ))}}\gg 1,\delta {{e}^{-\omega (\alpha -\pi (\centerdot ))}}\gg 1 \\ \end{aligned} \right. \\&\Rightarrow h(i,\omega )\approx \frac{N-i}{i+1}\times \frac{{{e}^{-\omega (\alpha -{{\pi }_{A}}(i+1))}}}{{{e}^{-\omega (\alpha -{{\pi }_{B}}(i))}}}\times \frac{\delta {{e}^{-\omega (\alpha -{{\pi }_{B}}(i))}}}{\delta {{e}^{-\omega (\alpha -{{\pi }_{A}}(i+1))}}}=\frac{N-i}{i+1} \\ \end{aligned}$$

On the basis of formula (25), it can be deduced that the corresponding average abundance function will basically remain unchanged at 1/2 when $r>4$. It can be seen from the above analysis that average abundance function will decreases when $r<3$. In addition, it increases when $3<r<4$ and basically remains unchanged at 1/2 when $r>4$.

#### The explanation for Corollary 5.2.3

Based on the characteristics of ${{\pi }_{A}}(i+1)$ and ${{\pi }_{B}}(i)$ ,it can be deduced that the inequality ${{\pi }_{B}}(i)<{{\pi }_{A}}(i+1)$ holds in most cases when $m=10$. This means that in the process of the increase of *r*, the ${{\pi }_{A}}(i+1)$ will become larger than aspiration level $\alpha$ and the ${{\pi }_{B}}(i)$ is still smaller than aspiration level $\alpha$ at the same time. In other words, ${{\pi }_{A}}(i+1)>\alpha$ and ${{\pi }_{B}}(i)< \alpha$. Therefore, more individuals will choose strategy *A*. So average abundance function will increase with *r*.

Furthermore, we will consider the situation when *r* keeps increasing ($r>4$). On the basis of formula (10a), it can be deduced that both ${{\pi }_{A}}(i+1)$ and ${{\pi }_{B}}(i)$ will increase to very high level when $r>4$. Moreover, both $\delta {{e}^{-\omega (\alpha -\pi \left( \centerdot \right) )}}$ and ${{e}^{-\omega (\alpha -\pi \left( \centerdot \right) )}}$ will increase to very high level when $r>4$. It can be seen from the above discussions that the following inequalities will be established:

25a $$\begin{aligned} {{e}^{-\omega (\alpha -\pi \left( \centerdot \right) )}}&>>1 \end{aligned}$$

25b $$\begin{aligned} \delta {{e}^{-\omega (\alpha -\pi \left( \centerdot \right) )}}&>>1 \end{aligned}$$

Then, on the basis of formula (25a), function $h\left( i,\omega \right)$ will gradually degenerate to the following expression:

26 $$\begin{aligned} &\left\{ \begin{aligned}&h(i,\omega )=\frac{N-i}{i+1}\times \frac{1+{{e}^{-\omega (\alpha -{{\pi }_{A}}(i+1))}}}{1+{{e}^{-\omega (\alpha -{{\pi }_{B}}(i))}}}\times \frac{1+\delta {{e}^{-\omega (\alpha -{{\pi }_{B}}(i))}}}{1+\delta {{e}^{-\omega (\alpha -{{\pi }_{A}}(i+1))}}} \\&{{e}^{-\omega (\alpha -\pi (\centerdot ))}}\gg 1,\delta {{e}^{-\omega (\alpha -\pi (\centerdot ))}}\gg 1 \\ \end{aligned} \right. \\&\Rightarrow h(i,\omega )\approx \frac{N-i}{i+1}\times \frac{{{e}^{-\omega (\alpha -{{\pi }_{A}}(i+1))}}}{{{e}^{-\omega (\alpha -{{\pi }_{B}}(i))}}}\times \frac{\delta {{e}^{-\omega (\alpha -{{\pi }_{B}}(i))}}}{\delta {{e}^{-\omega (\alpha -{{\pi }_{A}}(i+1))}}}=\frac{N-i}{i+1} \\ \end{aligned}$$

On the basis of formula (26), it can be deduced that the corresponding average abundance function will get close to 1/2 when $r>4$. In addition, we have already deduced that average abundance function is larger than 1/2 when $r<4$. Based on the above analysis, we can see that average abundance function will decrease (and get close to 1/2) when $r>4$.

It can be seen from the above analysis that average abundance function will increases when $r<4$. Moreover, it will decrease (and get close to 1/2) when $r>4$.

### The influence of *c* on average abundance function

**Fig. 12:**
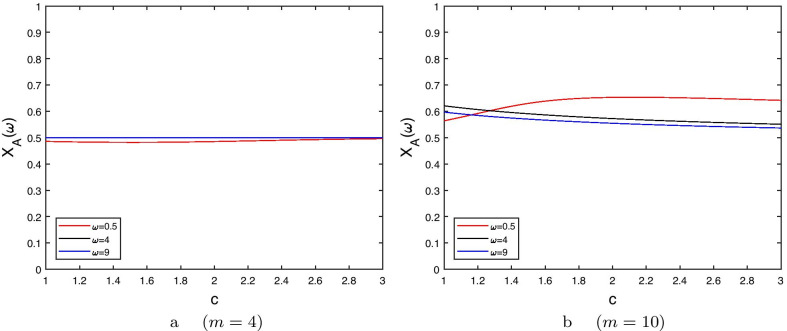
Average abundance function ${{X}_{A}}(\omega )$ changes with *c* ($d=15,r=8,\tau =0.25,\alpha =1,N=100$)

We can obtain Fig. 12 describing how average abundance function changes with *c*. Moreover, three corollaries can be obtained from the analysis of Fig. 12.

#### **Corollary 5.3.1**

*If *$m=4$, *average abundance function will decrease with* *c* when $\omega =0.5$.

#### **Corollary 5.3.2**

*If* $m=10$, *average abundance function will increase with* *c* *when* $\omega =0.5$.

#### **Corollary 5.3.3**

*If* $m=4$, *average abundance function will basically remain stable with the increase of* *c* *when *$\omega =4$ *or* $\omega =9$

#### **Corollary 5.3.4**

*If* $m=10$, *average abundance function will decrease (and get close to 1/2) with the increase of c* when $\omega =4$ *and* $\omega =9$.

The explanation for Corollary 5.3.1–5.3.4 is a little long and it is placed in Appendix B.

### The influence of $\alpha$ on average abundance function

**Fig. 13:**
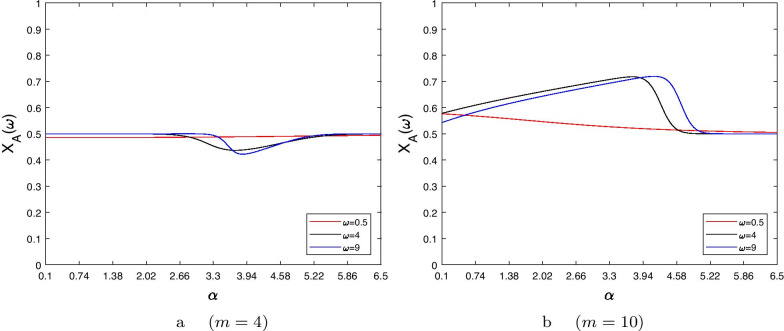
Average abundance function ${{X}_{A}}(\omega )$ changes with $\alpha$ ($d=15,r=8,c=1,\tau =0.25,N=100$)

We can obtain Fig. 13 describing how average abundance function changes with $\alpha$. Moreover, four corollaries can be obtained from the analysis of Fig. 13.

#### **Corollary 5.4.1**

*If* $m=4$, *average abundance function will basically remain stable with* $\alpha$ *when* $\omega =0.5$.

#### **Corollary 5.4.2**

*If *$m=10$, *average abundance function will decrease with* $\alpha$ *when* $\omega =0.5$.

#### **Corollary 5.4.3**

*If *$m=4$, *average abundance function will remain unchanged at 1/2 at first. Then it will decrease. After that it will increase. At last it will remain unchanged at 1/2 with the increase of *$\alpha$ *when* $\omega =4$ *and* $\omega =9$.

#### **Corollary 5.4.4**

*If* $m=10$, *average abundance function will increase at first. Then it will decrease. At last it will remain unchanged at 1/2 with the increase of* $\alpha$ *when* $\omega =4$ *and* $\omega =9$.

The explanation for Corollary 5.4.1-5.4.4 is a little long and it is placed in Appendix C.

### Brief summary

We have analyzed the influence of multiple parameters (*d*,*r*,*c* ,$\alpha$) on average abundance function with mutation ${X_A}(\omega )$ from both quantitative and qualitative aspects. Then we can obtain the following inferences:

#### Inference 5.1

The threshold *m* plays an important role in determining whether ${X_A}(\omega )<1/2$ or ${X_A}(\omega )>1/2$. In addition, the *m* will affect the influence of *d*,*r*,*c*,$\alpha$ on ${X_A}(\omega )$.

#### Inference 5.2

The influence of parameters *d*, *r*, *c* , $\alpha$ on average abundance function when selection intensity $\omega$ is small is slight.

#### Inference 6.3a

If $m=4$,average abundance function will decrease at first and then remain unchanged at 1/2 with the increase of *d*.

#### Inference 6.3b

If $m=10$,Average abundance function will decrease and get close to 1/2 with the increase of *d*.

#### Inference 6.4a

 If $m=4$,average abundance function will decrease at first ,and then increase,at last remain unchanged at 1/2 with the increase of *r*.

#### Inference 6.4b

If $m=10$,average abundance function will increase at first ,and then decrease with the increase of *r*.

#### Inference 6.5a

If $m=4$,average abundance function will basically remain stable with the increase of *c*.

#### Inference 6.5b

If $m=10$,average abundance function will decrease (and get close to 1/2) with the increase of *c*.

#### Inference 6.6a

If $m=4$,average abundance function will remain unchanged at 1/2 at first.Then it will decrease.After that it will increase.At last it will remain unchanged at 1/2 with the increase of $\alpha$.

#### Inference 6.6b

If $m=10$,average abundance function will increase at first.Then it will decrease.At last it will remain unchanged at 1/2 with the increase of $\alpha$.

## Conclusions

## The main research findings and prospects

### The main research findings of this article

The influences of cooperation and competition on social activities is becoming more and more important. This promotes the application of evolutionary game theory in real society and provides a good social background for us to study evolutionary game theory deeply.

Based on the study of literatures, the imitation rule, fixation probability, and structured population have been widely concerned and many results have been obtained. Relatively speaking, the research about the approximate expression of the average abundance function under different level of selection intensity deserves further discussion. Also how to use the theoretical deduction of average abundance function to explain the corresponding data simulation results is a field worth exploring. These will provide relevant research contents and research direction for this article. The main research findings are summarized as follows:

#### Conclusion 1

The approximate expression of average abundance function $X_{A}(\omega )$ when selection intensity $\omega$ is sufficient small ($\omega \rightarrow 0$) can be defined as follows:

$$\begin{aligned} {{X}_{A}}(\omega )= & {} \frac{1}{2}+\omega \frac{{1 - \delta }}{{1 + \delta }} \frac{1}{{{2}^{d+2}}}\frac{c}{d}\left[ \left( \frac{m\left( r-\tau \right) +d\tau }{r-\tau +d\tau }C_{d-1}^{m-1}+\sum \limits _{k=m}^{d-1}{C_{d-1}^{k}} \right) \left( r-\tau +d\tau \right) \right. \\&\left. -d\sum \limits _{k=m}^{d-1}{C_{d-1}^{k}} \right] \end{aligned}$$

#### Conclusion 2

The approximate expression of average abundance function $X_{A}(\omega )$ when selection intensity $\omega$ is large can be defined as follows:

$$\begin{aligned} X_{A}(\omega )= & {} {\frac{1}{N}}{\frac{\sum \limits _{j=1}^{N}{j{\prod \limits _{i=0}^{j-1}{h_a(i,\omega )}}}}{1+\sum \limits _{j=1}^{N}{\prod \limits _{i=0}^{j-1}{h_a(i,\omega )}}}} \\ {h_a}\left( {i,\omega } \right)= & {} \frac{{N - i}}{{i + 1}} \times \frac{{1 + \delta {e^{ - \omega \left( {\alpha - {\pi _B}\left( i \right) } \right) }}}}{{1 + \delta {e^{ - \omega \left( {\alpha - {\pi _A}\left( {i + 1} \right) } \right) }}}} \times {e^{ - \omega \left( {{\pi _B}\left( i \right) - {\pi _A}\left( {i + 1} \right) } \right) }} \end{aligned}$$

On one hand, approximation formula is not available when *r* is small and *m* is large. On the other hand, the approximation formula will become more reliable when $\omega$ is larger. In addition, the feasibility of approximate formula is the highest when *r* is large and *m* is small. Furthermore, function ${h_a}\left( {i,\omega } \right)$ can be divided into three parts. The first part $\frac{N-i}{i+1}$ essentially reflects that individuals are in neutral drift state. The second part $\frac{1+\delta {{e}^{-\omega (\alpha -{{\pi }_{B}}(i))}}}{1+\delta {{e}^{-\omega (\alpha -{{\pi }_{A}}(i+1))}}}$ is the inference term caused by mutation. The third part ${e^{ - \omega \left( {{\pi _B}\left( i \right) - {\pi _A}\left( {i + 1} \right) } \right) }}$ reflects the comparison between ${{\pi }_{A}}(i+1)$ and ${{\pi }_{B}}(i)$.

#### Conclusion 3

We analyze the effects of parameters (*d*,*r*,*c*,$\alpha$) on average abundance function $X_{A}(\omega )$ by numerical simulation and obtain Inference 6.1–6.6. In addition, the corresponding results have been explained based on the expected payoff function $\pi \left( \centerdot \right)$ and function $h(i,\omega )$.

### Research prospects

The characteristics of the average abundance function have been analyzed and some conclusions have been obtained in this article. However, the research work in this article is a preliminary exploration. It is necessary to conduct more research to improve the depth and breadth of this research. Further research can be carried out in the following aspects:

1. Based on conclusion 1, we can obtain the approximate expression of average abundance function $X_{A}(\omega )$ defined by formula (8) when selection intensity $\omega$ is sufficient small ($\omega \rightarrow 0$). Then we can analyze the applicability of formula (8) in the future research.
2. Based on conclusion 2, we can obtain the approximate expression of average abundance function $X_{A}(\omega )$ based on whether strategy *A* is favoured by selection or not when selection intensity $\omega$ is not too small. On the basis of formula (16), it can be concluded that *r*, *m*, *d*, and $\tau$ will play an important role in determining whether strategy *A* is favoured by selection or not. In the analysis of this article, we set the value of *d* and $\tau$ as fixed value and analyze the different values of *r* and *m*. This means we can analyze the effect of different values of *r*, *m*, *d*, and $\tau$ in the future research.
3. Based on conclusion 3, we analyze the effects of parameters (*d*,*r*,*c*,$\alpha$) on average abundance function $X_{A}(\omega )$ and obtain Inference 6.1-6.6. However, we only analyze the influence of single parameter in this situation. The joint influence of multiple parameters is still lacking. On the other hand, the sensitivity analysis of parameters is not enough. It means the above two fields should be taken seriously in the further study.
4. In addition, we have studied the characteristics of the average abundance function based on aspiration rule in finite well-mixed population. Corresponding analysis is still lacking for the research on other types of population (such as structured population) or other evolutionary rules (such as imitation rule). It means other types of population should be considered and other evolutionary rules should be taken into account.

## Data Availability

The data for this article can be found in 10.6084/m9.figshare.12559625.

## Appendix

### Appendix A The proof for property 5.1

We have already deduced that the approximate expression of average abundance function when selection intensity is sufficient small can be defined as follows:

27 $$\begin{aligned} {{X}_{A}}(\omega )= & {} \frac{1}{2}+\omega \frac{{1 - \delta }}{{1 + \delta }} \frac{1}{{{2}^{d+2}}}\frac{c}{d}\left[ \left( \frac{m\left( r-\tau \right) +d\tau }{r-\tau +d\tau }C_{d-1}^{m-1}+\sum \limits _{k=m}^{d-1}{C_{d-1}^{k}} \right) \left( r-\tau +d\tau \right) \right. \nonumber \\&\left. -d\sum \limits _{k=m}^{d-1}{C_{d-1}^{k}} \right] \end{aligned}$$

In addition, on the basis of formula (27), it can be deduced that:

28 $$\begin{aligned} &{{\left. {{X}_{A}}(\omega ) \right| }_{m+1}}-{{\left. {{X}_{A}}(\omega ) \right| }_{m}} \\&\quad =\left\{ \frac{1}{2}+\omega \frac{{1 - \delta }}{{1 + \delta }} \frac{1}{{{2}^{d+2}}}\frac{c}{d}\left[ \left( \frac{\left( m+1 \right) \left( r-\tau \right) +d\tau }{r-\tau +d\tau }C_{d-1}^{m} \right. \right. \right. \\&\left. \left. \left. \qquad +\sum \limits _{k=m+1}^{d-1}{C_{d-1}^{k}} \right) \left( r-\tau +d\tau \right) -d\sum \limits _{k=m+1}^{d-1}{C_{d-1}^{k}} \right] \right\} \\&\qquad -\left\{ \frac{1}{2}+\omega \frac{{1 - \delta }}{{1 + \delta }} \frac{1}{{{2}^{d+2}}}\frac{c}{d}\left[ \left( \frac{m\left( r-\tau \right) +d\tau }{r-\tau +d\tau }C_{d-1}^{m-1} \right. \right. \right. \\&\left. \left. \left. \qquad +\sum \limits _{k=m}^{d-1}{C_{d-1}^{k}} \right) \left( r-\tau +d\tau \right) -d\sum \limits _{k=m}^{d-1}{C_{d-1}^{k}} \right] \right\} \\&\quad =\omega \frac{{1 - \delta }}{{1 + \delta }} \frac{1}{{{2}^{d+2}}}\frac{c}{d}\left[ \left( \frac{\left( m+1 \right) \left( r-\tau \right) +d\tau }{r-\tau +d\tau }C_{d-1}^{m}-\frac{m\left( r-\tau \right) +d\tau }{r-\tau +d\tau }C_{d-1}^{m-1} \right. \right. \\&\left. \left. \qquad -C_{d-1}^{m} \right) \left( r-\tau +d\tau \right) +dC_{d-1}^{m} \right] \\&\quad =\omega \frac{{1 - \delta }}{{1 + \delta }} \frac{1}{{{2}^{d+2}}}\frac{c}{d}\frac{\left( d-1 \right) !}{\left( m-1 \right) !\left( d-1-m \right) !}\frac{-2\left( r-\tau \right) {{m}^{2}}+\left( r-2\tau -1 \right) dm+{{d}^{2}}}{m\left( d-m \right) } \\&\quad =\omega \frac{{1 - \delta }}{{1 + \delta }} \frac{1}{{{2}^{d+2}}}\frac{c}{d}\frac{\left( d-1 \right) !}{\left( m-1 \right) !\left( d-1-m \right) !}\frac{g\left( m \right) }{m\left( d-m \right) } \\ \end{aligned}$$

Inside the formula (28):

29 $$\begin{aligned} g\left( m \right) =-2\left( r-\tau \right) {{m}^{2}}+\left( r-2\tau -1 \right) dm+{{d}^{2}} \end{aligned}$$

Then, it can be deduced that the condition $0<m<d$ holds in multi-player threshold public goods evolutionary game model. Therefore, it can be deduced that whether formula (29) is positive or negative depends on function $g\left( m \right)$.In other words,whether ${{\left. {{X}_{A}}(\omega ) \right| }_{m+1}}-{{\left. {{X}_{A}}(\omega ) \right| }_{m}}$ is positive or negative will be determined by function $g\left( m \right)$.

Moreover, it can be deduced that function $g\left( m \right)$ is a monadic quadratic equation related to *m*. The two roots of this equation can be defined as follows:

30a $$\begin{aligned} {{m}_{1}}= & {} \frac{d}{4\left( r-\tau \right) }\left[ r-2\tau -1-\sqrt{{{\left( r-2\tau +3 \right) }^{2}}+8\left( \tau -1 \right) } \right] \end{aligned}$$

30b $$\begin{aligned} {{m}_{2}}= & {} \frac{d}{4\left( r-\tau \right) }\left[ r-2\tau -1+\sqrt{{{\left( r-2\tau +3 \right) }^{2}}+8\left( \tau -1 \right) } \right] \end{aligned}$$

On the basis of condition $r>1$, it can be deduced that:

30c $$\begin{aligned} {{m}_{1}}&=\frac{d}{4\left( r-\tau \right) }\left[ r-2\tau -1-\sqrt{{{\left( r-2\tau +3 \right) }^{2}}+8\left( \tau -1 \right) } \right] \nonumber \\&<\frac{d}{4\left( r-\tau \right) }\left[ r-2\tau -1-\sqrt{{{\left( r-2\tau -1 \right) }^{2}}} \right] =0 \end{aligned}$$

30d $$\begin{aligned} {{m}_{2}}&=\frac{d}{4\left( r-\tau \right) }\left[ r-2\tau -1+\sqrt{{{\left( r-2\tau +3 \right) }^{2}}+8\left( \tau -1 \right) } \right] \nonumber \\&<\frac{d}{4\left( r-\tau \right) }\left[ r-2\tau -1+\sqrt{9{{r}^{2}}-12r\tau +4{{\tau }^{2}}+6r-4\tau +1} \right] \nonumber \\&=\frac{d}{4\left( r-\tau \right) }\left[ r-2\tau -1+3r-2\tau +1 \right] \nonumber \\&=\frac{d}{4\left( r-\tau \right) }\left[ 4r-4\tau \right] =d \end{aligned}$$

Combined with the above analysis, we can see that ${{m}_{1}}<0$ and $0<{{m}_{2}}<d$. Furthermore, the ${{m}_{1}}$ will be discarded and ${{m}_{2}}$ will be retained because the condition $0<m<d$ should be satisfied in threshold public goods evolutionary game model.

Then, on the basis of the properties of monadic quadratic equation, it can be deduced that function $g\left( m \right)$ is larger than 0 when $0<m<{{m}_{2}}$. On the other hand, function $g\left( m \right)$ is smaller than 0 when ${{m}_{2}}<m<d$. In addition, function $g\left( m \right)$ will be equal to 0 when $m={{m}_{2}}$. Combined with the above analysis, we can see that:

31 $$\begin{aligned} g\left( m \right) =\left\{ \begin{matrix} >0,\begin{array}{*{35}{l}} {} \\ \end{array}\begin{array}{*{35}{l}} {} \\ \end{array}\begin{array}{*{35}{l}} {} \\ \end{array}\begin{array}{*{35}{l}} {} \\ \end{array}0<m<{{m}_{2}} \\ =0,\begin{array}{*{35}{l}} \begin{array}{*{35}{l}} {} \\ \end{array} \\ \end{array}\begin{array}{*{35}{l}} {} \\ \end{array}\begin{array}{*{35}{l}} {} \\ \end{array}\begin{array}{*{35}{l}} {} \\ \end{array}\begin{array}{*{35}{l}} {} \\ \end{array}\begin{array}{*{35}{l}} {} \\ \end{array}m={{m}_{2}} \\<0,\begin{array}{*{35}{l}} {} \\ \end{array}\begin{array}{*{35}{l}} {} \\ \end{array}\begin{array}{*{35}{l}} {} \\ \end{array}\begin{array}{*{35}{l}} {} \\ \end{array}{{m}_{2}}<m<d \\ \end{matrix} \right. \end{aligned}$$

It has been deduced that whether ${{\left. {{X}_{A}}(\omega ) \right| }_{m+1}}-{{\left. {{X}_{A}}(\omega ) \right| }_{m}}$ is positive or negative will be determined by function $g\left( m \right)$. Then it can be deduced that:

32 $$\begin{aligned} {{\left. {{X}_{A}}(\omega ) \right| }_{m+1}}-{{\left. {{X}_{A}}(\omega ) \right| }_{m}}=\left\{ \begin{matrix} >0,\begin{array}{*{35}{l}} {} \\ \end{array}\begin{array}{*{35}{l}} {} \\ \end{array}0<m<{{m}_{2}} \\ =0,\begin{array}{*{35}{l}} {} \\ \end{array}\begin{array}{*{35}{l}} {} \\ \end{array}\begin{array}{*{35}{l}} {} \\ \end{array}\begin{array}{*{35}{l}} {} \\ \end{array}m={{m}_{2}} \\<0,\begin{array}{*{35}{l}} {} \\ \end{array}\begin{array}{*{35}{l}} {} \\ \end{array}{{m}_{2}}<m<d \\ \end{matrix} \right. \end{aligned}$$

On the basis of formula (32), it can be deduced that ${{X}_{A}}(\omega )$ increases with *m* when $0<m<{{m}_{2}}$. On the other hand, ${{X}_{A}}(\omega )$ decreases when ${{m}_{2}}+1<m<d$. In addition, ${{X}_{A}}(\omega )$ will reach the maximum value if $m={{m}_{2}}$ or $m={{m}_{2}}+1$.

Moreover, it can be deduced that ${{m}_{2}}$ is a decimal. However, *m* must be an integer. On the basis of this finding, it can be deduced that ${{X}_{A}}(\omega )$ will reach the maximum value when $m={\text {int}}\left\{ \frac{d}{4\left( r-\tau \right) }\left[ r-2\tau -1+\sqrt{{{\left( r-2\tau +3 \right) }^{2}}+8\left( \tau -1 \right) } \right] \right\} +1$.

### Appendix B The explanation for Corollary 5.3.1-5.3.4

#### The explanation for Corollary 5.3.1

The corollary 5.3.1 can be explained based on formula (17):

$$\begin{aligned} {X_A}\left( \omega \right)= & {} \frac{1}{2} + \omega \frac{{1 - \delta }}{{1 + \delta }} \frac{1}{{{2^{d + 2}}}}\frac{c}{d}\left[ \left( \frac{{m\left( {r - \tau } \right) + d\tau }}{{r - \tau + d\tau }}C_{d - 1}^{m - 1} + \sum \limits _{k = m}^{d - 1} {C_{d - 1}^k} \right) \left( r - \tau \right. \right. \\&\left. \left. + d\tau \right) - d\sum \limits _{k = m}^{d - 1} {C_{d - 1}^k} \right] \end{aligned}$$

Based on formula (17), it can be deduced that:

33 $$\begin{aligned}&\frac{{\partial {X_A}\left( \omega \right) }}{{\partial c}} \nonumber \\&\quad = \omega \frac{{1 - \delta }}{{1 + \delta }} \frac{1}{{{2^{d + 2}}}}\frac{1}{d}\left[ \left( {\frac{{m\left( {r - \tau } \right) + d\tau }}{{r - \tau + d\tau }}C_{d - 1}^{m - 1} + \sum \limits _{k = m}^{d - 1} {C_{d - 1}^k} } \right) \left( r - \tau \right. \right. \nonumber \\&\left. \left. \qquad + d\tau \right) - d\sum \limits _{k = m}^{d - 1} {C_{d - 1}^k} \right] \nonumber \\&\quad =\omega \frac{{1 - \delta }}{{1 + \delta }} \frac{1}{{{2^{d + 2}}}}\frac{1}{d}\left[ {\left( {m\left( {r - \tau } \right) + d\tau } \right) C_{d - 1}^{m - 1} + \left( {r - \tau + d\tau - d} \right) \sum \limits _{k = m}^{d - 1} {C_{d - 1}^k} } \right] \end{aligned}$$

When $m=4$, by inserting the values of parameters into the $\frac{{\partial {X_A}(\omega )}}{{\partial c}}$, it can be deduced that:

34 $$\begin{aligned} \left( {m\left( {r - \tau } \right) + d\tau } \right) C_{d - 1}^{m - 1} + \left( {r - \tau + d\tau - d} \right) \sum \limits _{k = m}^{d - 1} {C_{d - 1}^k} = \frac{{139}}{4}C_{14}^3 - \frac{{14}}{4}\sum \limits _{k = 4}^{14} {C_{14}^k} \end{aligned}$$

Based on the properties of permutation and combination function, the following inequality will hold:

35 $$\begin{aligned} 14\sum \limits _{k = 4}^{14} {C_{14}^k}> 612C_{14}^3 > 139C_{14}^3 \end{aligned}$$

Based on formulas (33)–(35), it can be deduced that $\frac{{\partial {X_A}(\omega )}}{{\partial c}} < 0$. So average abundance function will decrease with *c* when $\omega =0.5$. In addition, the trend is close to linear change.

#### The explanation for Corollary 5.3.2

When $m=10$, by inserting the values of parameters into the $\frac{{\partial {X_A}(\omega )}}{{\partial c}}$, it can be deduced that:

36 $$\begin{aligned} \left( {m\left( {r - \tau } \right) + d\tau } \right) C_{d - 1}^{m - 1} + \left( {r - \tau + d\tau - d} \right) \sum \limits _{k = m}^{d - 1} {C_{d - 1}^k} = \frac{{325}}{4}C_{14}^9 - \frac{{14}}{4}\sum \limits _{k = 10}^{14} {C_{14}^k} \end{aligned}$$

Based on the properties of permutation and combination function, the following inequality will hold:

37 $$\begin{aligned} 14\sum \limits _{k = 10}^{14} {C_{14}^k}< 11C_{14}^9 < 325C_{14}^9 \end{aligned}$$

Based on formulas (36)–(37), it can be deduced that $\frac{{\partial {X_A}(\omega )}}{{\partial c}} > 0$. So average abundance function will increase with *c* when $\omega =0.5$.

It should be noted the corresponding trend is not linear change when $m=10$. Furthermore, it can be deduced that the applicability of formula (17) is limited when $m=10$. It indicates that the complete applicability of formula (17) requires that *m* should be smaller (at lest $m<10$).

#### The explanation for Corollary 5.3.3

The main point of analysis lies in the expected payoff function $\pi \left( \centerdot \right)$ defined by formula (10a) and function $h\left( i,\omega \right)$ defined by formula (7a).

On the basis of formula (10a), the derivative of ${{\pi }_{A}}(i+1)$ and ${{\pi }_{B}}(i)$ with respect to *c* can be defined as follows:

37a $$\begin{aligned} \frac{{\partial {\pi _A}\left( {i + 1} \right) }}{{\partial c}}= & {} \frac{{\partial \left[ {\sum \limits _{k = m - 1}^{d - 1} {\frac{{C_i^kC_{N - i - 1}^{d - 1 - k}}}{{C_{N - 1}^{d - 1}}}} \left( {\frac{{k + 1}}{d}\left( {r - \tau } \right) c + \tau c} \right) } \right] }}{{\partial c}}\nonumber \\= & {} \sum \limits _{k = m - 1}^{d - 1} {\frac{{C_i^kC_{N - i - 1}^{d - 1 - k}}}{{C_{N - 1}^{d - 1}}}} \left( {\frac{{k + 1}}{d}\left( {r - \tau } \right) + \tau } \right) \end{aligned}$$

37b $$\begin{aligned} \frac{{\partial {\pi _B}\left( i \right) }}{{\partial c}}= & {} \frac{{\partial \left[ {\sum \limits _{k = m}^{d - 1} {\frac{{C_i^kC_{N - i - 1}^{d - 1 - k}}}{{C_{N - 1}^{d - 1}}}} \left( {\frac{k}{d}\left( {r - \tau } \right) c + c} \right) } \right] }}{{\partial c}}\nonumber \\= & {} \sum \limits _{k = m}^{d - 1} {\frac{{C_i^kC_{N - i - 1}^{d - 1 - k}}}{{C_{N - 1}^{d - 1}}}} \left( {\frac{k}{d}\left( {r - \tau } \right) + 1} \right) \end{aligned}$$

Then,on the basis of formula (B.6), it can be deduced that both ${{\pi }_{A}}(i+1)$ and ${{\pi }_{B}}(i)$ will increase with *c*. In addition, by analyzing the characteristics of ${{\pi }_{A}}(i+1)$ and ${{\pi }_{B}}(i)$, it can be deduced that both ${{\pi }_{A}}(i+1)$ and ${{\pi }_{B}}(i)$ will increase to very high level when *r* is large($r=8$).

Combined with the above discussion, we can see that both ${{\pi }_{A}}(i+1)$ and ${{\pi }_{B}}(i)$ are larger than aspiration level $\alpha$. In other words, ${{\pi }_{A}}(i+1)>\alpha$, ${{\pi }_{B}}(i)>\alpha$. Furthermore, both $\delta {{e}^{-\omega (\alpha -\pi \left( \centerdot \right) )}}$ and ${{e}^{-\omega (\alpha -\pi \left( \centerdot \right) )}}$ will increase to very high level. It can be seen from the above discussions that the following inequalities will be established:

37c $$\begin{aligned} {{e}^{-\omega (\alpha -\pi \left( \centerdot \right) )}}&>>1 \end{aligned}$$

37d $$\begin{aligned} \delta {{e}^{-\omega (\alpha -\pi \left( \centerdot \right) )}}&>>1 \end{aligned}$$

Then, on the basis of formula (B.7), function $h\left( i,\omega \right)$ will gradually degenerate to the following expression:

38 $$\begin{aligned} &\left\{ \begin{aligned}&h(i,\omega )=\frac{N-i}{i+1}\times \frac{1+{{e}^{-\omega (\alpha -{{\pi }_{A}}(i+1))}}}{1+{{e}^{-\omega (\alpha -{{\pi }_{B}}(i))}}}\times \frac{1+\delta {{e}^{-\omega (\alpha -{{\pi }_{B}}(i))}}}{1+\delta {{e}^{-\omega (\alpha -{{\pi }_{A}}(i+1))}}} \\&{{e}^{-\omega (\alpha -\pi (\centerdot ))}}\gg 1,\delta {{e}^{-\omega (\alpha -\pi (\centerdot ))}}\gg 1 \\ \end{aligned} \right. \\&\quad \Rightarrow h(i,\omega )\approx \frac{N-i}{i+1}\times \frac{{{e}^{-\omega (\alpha -{{\pi }_{A}}(i+1))}}}{{{e}^{-\omega (\alpha -{{\pi }_{B}}(i))}}}\times \frac{\delta {{e}^{-\omega (\alpha -{{\pi }_{B}}(i))}}}{\delta {{e}^{-\omega (\alpha -{{\pi }_{A}}(i+1))}}}=\frac{N-i}{i+1} \\ \end{aligned}$$

On the basis of formula (38), it can be deduced that the corresponding average abundance function will basically remain unchanged at 1/2 when $r>4$. It can be seen from the above analysis that average abundance function basically remains unchanged at 1/2.

#### The explanation for Corollary 5.3.4

Similar to the analysis for corollary 5.3.3, it can be concluded that average abundance function will get close to 1/2 when $m=10$.

However, it can be concluded that there must be more collaborators in the group to ensure that threshold public goods game can continue when *m* increase($m=4$ increases to $m=10$). Therefore, average abundance function when $m=10$ is larger than average abundance function when $m=4$. Then it can be seen from the above analysis that average abundance function is larger than 1/2 when $m=10$.

We have already deduced that average abundance function will get close to 1/2. In addition, average abundance function is larger than 1/2. Furthermore, it can be concluded that average abundance function will decrease (and get close to 1/2) with the increase of *c* when $m=10$.

### Appendix C The explanation for Corollary 5.4.1-5.4.4

#### The explanation for Corollary 5.4.1

The corollary 5.4.1 can be explained based on formula (17):

$$\begin{aligned} {X_A}\left( \omega \right)= & {} \frac{1}{2} + \omega \frac{{1 - \delta }}{{1 + \delta }} \frac{1}{{{2^{d + 2}}}}\frac{c}{d}\left[ \left( {\frac{{m\left( {r - \tau } \right) + d\tau }}{{r - \tau + d\tau }}C_{d - 1}^{m - 1} + \sum \limits _{k = m}^{d - 1} {C_{d - 1}^k} } \right) \left( r - \tau \right. \right. \\&\left. \left. + d\tau \right) - d\sum \limits _{k = m}^{d - 1} {C_{d - 1}^k} \right] \end{aligned}$$

Based on formula (17), it can be deduced that $\frac{{\partial {X_A}(\omega )}}{{\partial \alpha }} = 0$. So average abundance function will basically remain stable with the increase of $\alpha$ when $\omega =0.5$ if $m=4$.

#### The explanation for Corollary 5.4.2

It should be noted that average abundance function will decrease with $\alpha$ when $\omega =0.5$ if $m=10$. This phenomenon is inconsistent with the conclusion that $\frac{{\partial {X_A}(\omega )}}{{\partial \alpha }} = 0$. Furthermore, it can be deduced that the applicability of formula (17) is limited when $m=10$. It indicates that the complete applicability of formula (17) requires that *m* should be smaller (at lest $m<10$).

#### The explanation for Corollary 5.4.3

The main point of analysis lies in the expected payoff function $\pi \left( \centerdot \right)$ defined by formula (10a) and function $h\left( i,\omega \right)$ defined by formula (7a).

On the basis of formula (10a), the derivative of ${{\pi }_{A}}(i+1)$ and ${{\pi }_{B}}(i)$ with respect to $\alpha$ can be defined as follows:

38a $$\begin{aligned} \frac{{\partial {\pi _A}\left( {i + 1} \right) }}{{\partial \alpha }}= & {} \frac{{\partial \left[ {\sum \limits _{k = m - 1}^{d - 1} {\frac{{C_i^kC_{N - i - 1}^{d - 1 - k}}}{{C_{N - 1}^{d - 1}}}} \left( {\frac{{k + 1}}{d}\left( {r - \tau } \right) c + \tau c} \right) } \right] }}{{\partial \alpha }} = 0 \end{aligned}$$

38b $$\begin{aligned} \frac{{\partial {\pi _B}\left( i \right) }}{{\partial \alpha }}= & {} \frac{{\partial \left[ {\sum \limits _{k = m}^{d - 1} {\frac{{C_i^kC_{N - i - 1}^{d - 1 - k}}}{{C_{N - 1}^{d - 1}}}} \left( {\frac{k}{d}\left( {r - \tau } \right) c + c} \right) } \right] }}{{\partial \alpha }} = 0 \end{aligned}$$

Then, on the basis of formula (C.1), it can be deduced that neither ${{\pi }_{A}}(i+1)$ nor ${{\pi }_{B}}(i)$ is related to $\alpha$.

At first, we will consider the situation when $\alpha <2.66$. It can be deduced that when $\alpha$ is small ($\alpha <2.66$), both ${{\pi }_{A}}(i+1)$ and ${{\pi }_{B}}(i)$ are larger than aspiration level $\alpha$. In other words, there will be ${{\pi }_{B}}(i)>\alpha$ and ${{\pi }_{A}}(i+1)>\alpha$ when $\alpha <2.66$. Moreover, both $\delta {{e}^{-\omega (\alpha -\pi \left( \centerdot \right) )}}$ and ${{e}^{-\omega (\alpha -\pi \left( \centerdot \right) )}}$ will increase to very high level when $\alpha <2.66$. It can be seen from the above discussions that the following inequalities will be established:

38c $$\begin{aligned} {{e}^{-\omega (\alpha -\pi \left( \centerdot \right) )}}&>>1 \end{aligned}$$

38d $$\begin{aligned} \delta {{e}^{-\omega (\alpha -\pi \left( \centerdot \right) )}}&>>1 \end{aligned}$$

Then, on the basis of formula (C.2), function $h\left( i,\omega \right)$ will gradually degenerate to the following expression:

39 $$\begin{aligned} &\left\{ \begin{aligned}&h(i,\omega )=\frac{N-i}{i+1}\times \frac{1+{{e}^{-\omega (\alpha -{{\pi }_{A}}(i+1))}}}{1+{{e}^{-\omega (\alpha -{{\pi }_{B}}(i))}}}\times \frac{1+\delta {{e}^{-\omega (\alpha -{{\pi }_{B}}(i))}}}{1+\delta {{e}^{-\omega (\alpha -{{\pi }_{A}}(i+1))}}} \\&{{e}^{-\omega (\alpha -\pi (\centerdot ))}}\gg 1,\delta {{e}^{-\omega (\alpha -\pi (\centerdot ))}}\gg 1 \\ \end{aligned} \right. \\&\quad \Rightarrow h(i,\omega )\approx \frac{N-i}{i+1}\times \frac{{{e}^{-\omega (\alpha -{{\pi }_{A}}(i+1))}}}{{{e}^{-\omega (\alpha -{{\pi }_{B}}(i))}}}\times \frac{\delta {{e}^{-\omega (\alpha -{{\pi }_{B}}(i))}}}{\delta {{e}^{-\omega (\alpha -{{\pi }_{A}}(i+1))}}}=\frac{N-i}{i+1} \\ \end{aligned}$$

On the basis of formula (39), it can be deduced that the corresponding average abundance function will basically remain unchanged at 1/2 when $\alpha <2.66$.

Then, we will consider the situation when $\alpha >2.66$. It has been deduced that neither ${{\pi }_{A}}(i+1)$ nor ${{\pi }_{B}}(i)$ is related to $\alpha$. In addition, by analyzing the characteristics of ${{\pi }_{A}}(i+1)$ and ${{\pi }_{B}}(i)$, it can be deduced that the inequality ${{\pi }_{B}}(i)>{{\pi }_{A}}(i+1)$ holds in most cases when $m=4$.

On the whole, it can be deduced that when $\alpha$ keeps increasing ($\alpha >2.66$), the ${{\pi }_{A}}(i+1)$ will become smaller than aspiration level $\alpha$, while the ${{\pi }_{B}}(i)$ is still larger than aspiration level $\alpha$ at the same time. In other words, there will be ${{\pi }_{A}}(i+1)<\alpha$ and ${{\pi }_{B}}(i)>\alpha$ when $\alpha$ keeps increasing. Therefore, average abundance function will decrease when $\alpha >2.66$.

In addition, we will consider the situation when $\alpha >3.3$. It can be deduced that both ${{\pi }_{A}}(i+1)$ and ${{\pi }_{B}}(i)$ will become smaller than aspiration level $\alpha$ when $\alpha$ increases to high level ($\alpha >3.3$). In other words, ${{\pi }_{A}}(i+1)<\alpha$, ${{\pi }_{B}}(i)<\alpha$. It can be seen from the above discussions that the following inequalities will be established:

40a $$\begin{aligned} {{e}^{-\omega (\alpha -\pi \left( \centerdot \right) )}}&<<1 \end{aligned}$$

40b $$\begin{aligned} \delta {{e}^{-\omega (\alpha -\pi \left( \centerdot \right) )}}&<<1 \end{aligned}$$

Then, on the basis of formula (C.4), function $h\left( i,\omega \right)$ will gradually degenerate to the following expression:

41 $$\begin{aligned}&\left\{ \begin{array}{lr} h\left( i,\omega \right) =\frac{N-i}{i+1}\times \frac{1+{{e}^{-\omega (\alpha -{{\pi }_{A}}(i+1))}}}{1+{{e}^{-\omega (\alpha -{{\pi }_{B}}(i))}}}\times \frac{1+\delta {{e}^{-\omega (\alpha -{{\pi }_{B}}(i))}}}{1+\delta {{e}^{-\omega (\alpha -{{\pi }_{A}}(i+1))}}} &{} \\ {{e}^{-\omega (\alpha -\pi \left( \varvec{\cdot } \right) )}}<<1,\delta {{e}^{-\omega (\alpha -\pi \left( \varvec{\cdot } \right) )}}<<1 &{} \\ \end{array} \right. \nonumber \\&\quad \Rightarrow h\left( i,\omega \right) \approx \frac{N-i}{i+1}\times \frac{\begin{array}{*{35}{l}} \begin{array}{*{35}{l}} 1 \\ \end{array} \\ \end{array}}{\begin{array}{*{35}{l}} 1 \\ \end{array}}\times \frac{\begin{array}{*{35}{l}} \begin{array}{*{35}{l}} 1 \\ \end{array} \\ \end{array}}{\begin{array}{*{35}{l}} 1 \\ \end{array}}=\frac{N-i}{i+1} \end{aligned}$$

On the basis of formula (41), it can be deduced that the corresponding average abundance function will get close to 1/2 when $\alpha >3.3$. In addition, we have obtained that average abundance function is smaller than 1/2 when $\alpha <3.3$. Based on the above analysis, we can see that average abundance function will increases (and get close to 1/2) when $\alpha >3.3$.

At last, it can be easily deduced that average abundance function will reach 1/2 when $\alpha$ keeps increasing to very high level($\alpha >5.22$). Furthermore, there is a balance between the proportion of individuals choosing strategy *A* and the proportion of individuals choosing strategy *B* at this time.

It can be seen from the above analysis that average abundance function will remain unchanged at 1/2 when $\alpha <2.66$. In addition, it will decrease when $2.66<\alpha <3.3$. Moreover, it will increase when $3.3<\alpha <5.22$. At last, it will remain unchanged at 1/2 when $\alpha >5.22$.

#### The explanation for Corollary 5.4.4

The main point of analysis lies in the expected payoff function $\pi \left( \centerdot \right)$ defined by formula (10a) and function $h\left( i,\omega \right)$ defined by formula (7a).

At first, we will consider the situation when $\alpha <3.94$. It can be deduced that both ${{e}^{-\omega (\alpha -\pi \left( \centerdot \right) )}}$ and $\delta {{e}^{-\omega (\alpha -\pi \left( \centerdot \right) )}}$ will decrease with $\alpha$. In addition, it can be obtained that the decreasing rate of $\delta {{e}^{-\omega (\alpha -\pi \left( \centerdot \right) )}}$ is much larger than that of ${{e}^{-\omega (\alpha -\pi \left( \centerdot \right) )}}$ because of the existence of mutation $\delta$. For example, the decreasing rate of $\delta {{e}^{-\omega (\alpha -\pi \left( \centerdot \right) )}}$ is 32 times larger than that of ${{e}^{-\omega (\alpha -\pi \left( \centerdot \right) )}}$ when $\delta =0.03$.

It can be seen from the above discussions that the following inequalities will be established when $m=10$:

41a $$\begin{aligned} {{e}^{-\omega (\alpha -\pi \left( \centerdot \right) )}}&<1 \end{aligned}$$

41b $$\begin{aligned} \delta {{e}^{-\omega (\alpha -\pi \left( \centerdot \right) )}}&<<1 \end{aligned}$$

Then, on the basis of formula (C.6), function $h\left( i,\omega \right)$ will gradually degenerate to the following expression:

42 $$\begin{aligned} &\left\{ \begin{matrix} h\left( i,\omega \right) =\frac{N-i}{i+1}\times \frac{1+{{e}^{-\omega (\alpha -{{\pi }_{A}}(i+1))}}}{1+{{e}^{-\omega (\alpha -{{\pi }_{B}}(i))}}}\times \frac{1+\delta {{e}^{-\omega (\alpha -{{\pi }_{B}}(i))}}}{1+\delta {{e}^{-\omega (\alpha -{{\pi }_{A}}(i+1))}}} \\ \delta {{e}^{-\omega (\alpha -\pi \left( \centerdot \right) )}}<<1 \\ \end{matrix} \right. \\&\quad \Rightarrow h\left( i,\omega \right) \approx \frac{N-i}{i+1}\times \frac{1+{{e}^{-\omega (\alpha -{{\pi }_{A}}(i+1))}}}{1+{{e}^{-\omega (\alpha -{{\pi }_{B}}(i))}}}\times \frac{\begin{array}{*{35}{l}} \begin{array}{*{35}{l}} 1 \\ \end{array} \\ \end{array}}{\begin{array}{*{35}{l}} 1 \\ \end{array}}=\frac{N-i}{i+1}\times \frac{1+{{e}^{-\omega (\alpha -{{\pi }_{A}}(i+1))}}}{1+{{e}^{-\omega (\alpha -{{\pi }_{B}}(i))}}} \\ \end{aligned}$$

Combined with the above discussion, we can see that $h\left( i,\omega \right) \approx \frac{N-i}{i+1}\times \frac{1+{{e}^{-\omega (\alpha -{{\pi }_{A}}(i+1))}}}{1+{{e}^{-\omega (\alpha -{{\pi }_{B}}(i))}}}$ when $m=10$. Therefore, we will set $h\left( i,\omega \right) =\frac{N-i}{i+1}\times \frac{1+{{e}^{-\omega (\alpha -{{\pi }_{A}}(i+1))}}}{1+{{e}^{-\omega (\alpha -{{\pi }_{B}}(i))}}}$ in the following discussion.

At the beginning, we suppose that function $h\left( i,\omega \right)$ will change to $\overline{h\left( i,\omega \right) }$ when aspiration level $\alpha$ increases to $\alpha \text {+}\beta$ ($\beta >0$). Then,it can be deduced that the most important problem is to compare $h\left( i,\omega \right)$ with $\overline{h\left( i,\omega \right) }$. Furthermore, The difference between between $h\left( i,\omega \right)$ and $\overline{h\left( i,\omega \right) }$ can be defined as follows:

43 $$\begin{aligned} &\overline{h\left( i,\omega \right) }-h\left( i,\omega \right) \\&\quad =\frac{N-i}{i+1}\times \frac{1+{{e}^{-\omega (\alpha +\beta -{{\pi }_{A}}(i+1))}}}{1+{{e}^{-\omega (\alpha +\beta -{{\pi }_{B}}(i))}}}-\frac{N-i}{i+1}\times \frac{1+{{e}^{-\omega (\alpha -{{\pi }_{A}}(i+1))}}}{1+{{e}^{-\omega (\alpha -{{\pi }_{B}}(i))}}} \\&\quad =\frac{N-i}{i+1}\times \left[ \frac{{{e}^{-\omega (\alpha -{{\pi }_{B}}(i))}}\text {+}{{e}^{-\omega (\alpha +\beta -{{\pi }_{A}}(i+1))}}-{{e}^{-\omega (\alpha +\beta -{{\pi }_{B}}(i))}}-{{e}^{-\omega (\alpha -{{\pi }_{A}}(i+1))}}}{\left( 1+{{e}^{-\omega (\alpha +\beta -{{\pi }_{B}}(i))}} \right) \times \left( 1+{{e}^{-\omega (\alpha -{{\pi }_{B}}(i))}} \right) } \right] \\&\quad =\frac{N-i}{i+1}\times \left[ \frac{{{e}^{-\omega (\alpha -{{\pi }_{B}}(i))}}\left( 1-{{e}^{-\omega \beta }} \right) \text {+}{{e}^{-\omega (\alpha -{{\pi }_{A}}(i+1))}}\left( {{e}^{-\omega \beta }}-1 \right) }{\left( 1+{{e}^{-\omega (\alpha +\beta -{{\pi }_{B}}(i))}} \right) \times \left( 1+{{e}^{-\omega (\alpha -{{\pi }_{B}}(i))}} \right) } \right] \\&\quad =\frac{N-i}{i+1}\times \left[ \frac{\left( 1-{{e}^{-\omega \beta }} \right) {{e}^{-\omega \alpha }}\left( {{e}^{\omega {{\pi }_{B}}(i)}}-{{e}^{\omega {{\pi }_{A}}(i+1)}} \right) }{\left( 1+{{e}^{-\omega (\alpha +\beta -{{\pi }_{B}}(i))}} \right) \times \left( 1+{{e}^{-\omega (\alpha -{{\pi }_{B}}(i))}} \right) } \right] \\ \end{aligned}$$

It has been deduced that the inequality ${{\pi }_{B}}(i)>{{\pi }_{A}}(i+1)$ holds. Then, we can obtain that ${{e}^{\omega {{\pi }_{B}}(i)}}>{{e}^{\omega {{\pi }_{A}}(i+1)}}$. In addition, it can be deduced that ${{e}^{-\omega \beta }}<1$ because $\beta >0$. It can be seen from the above discussions that the following inequality will be established:

44 $$\begin{aligned} &\left\{ \begin{matrix} {{e}^{\omega {{\pi }_{B}}(i)}}>{{e}^{\omega {{\pi }_{A}}(i+1)}} \\ {{e}^{-\omega \beta }}<1 \\ \end{matrix} \right. \\&\quad \Rightarrow \overline{h\left( i,\omega \right) }-h\left( i,\omega \right) =\frac{N-i}{i+1}\times \left[ \frac{\left( 1-{{e}^{-\omega \beta }} \right) {{e}^{-\omega \alpha }}\left( {{e}^{\omega {{\pi }_{B}}(i)}}-{{e}^{\omega {{\pi }_{A}}(i+1)}} \right) }{\left( 1+{{e}^{-\omega (\alpha +\beta -{{\pi }_{B}}(i))}} \right) \times \left( 1+{{e}^{-\omega (\alpha -{{\pi }_{B}}(i))}} \right) } \right] >0 \\ \end{aligned}$$

On the basis of formula (44), it can be deduced that function $h\left( i,\omega \right)$ will increase with $\alpha$. Then it can be concluded that the average abundance function will increase with $\alpha$ when $\alpha <3.94$.

Then, we will consider the situation when $\alpha >3.94$. It can be deduced that both ${{\pi }_{A}}(i+1)$ and ${{\pi }_{B}}(i)$ will be smaller than aspiration level $\alpha$ when $\alpha >3.94$. In other words, ${{\pi }_{A}}(i+1)<\alpha$,${{\pi }_{B}}(i)<\alpha$. Furthermore, both $\delta {{e}^{-\omega (\alpha -\pi \left( \centerdot \right) )}}$ and ${{e}^{-\omega (\alpha -\pi \left( \centerdot \right) )}}$ will decrease to very low level. It can be seen from the above discussions that the following inequalities will be established:

45 $$\begin{aligned} {{e}^{-\omega (\alpha -\pi \left( \centerdot \right) )}}<<1 \end{aligned}$$

Then, on the basis of formula (45), function $h\left( i,\omega \right)$ will gradually degenerate to the following expression:

46 $$\begin{aligned} &\left\{ \begin{matrix} h\left( i,\omega \right) =\frac{N-i}{i+1}\times \frac{1+{{e}^{-\omega (\alpha -{{\pi }_{A}}(i+1))}}}{1+{{e}^{-\omega (\alpha -{{\pi }_{B}}(i))}}} \\ {{e}^{-\omega (\alpha -\pi \left( \centerdot \right) )}}<<1 \\ \end{matrix} \right. \\&\quad \Rightarrow h\left( i,\omega \right) \approx \frac{N-i}{i+1}\times \frac{\begin{array}{*{35}{l}} \begin{array}{*{35}{l}} 1 \\ \end{array} \\ \end{array}}{\begin{array}{*{35}{l}} 1 \\ \end{array}}=\frac{N-i}{i+1} \\ \end{aligned}$$

Therefore, the corresponding average abundance function will get close to 1/2 when $\alpha$ increases to a very high level ($\alpha >3.94$ ). In addition, it has been deduced that average abundance function is larger than 1/2 when $\alpha <3.94$. Based on the above analysis, we can see that average abundance function will decrease (and get close to 1/2) when $\alpha >3.94$.

Then, it can be easily deduced that average abundance function will reach 1/2 when $\alpha$ keeps increasing to very high level($\alpha >4.58$). Furthermore, there is a balance between the proportion of individuals choosing strategy *A* and the proportion of individuals choosing strategy *B* at this time.

Combined with the above discussion, we can see that average abundance function will increase when $\alpha <3.94$. In addition, it will decrease when $3.94<\alpha <4.58$. At last, it will remain unchanged at 1/2 when $\alpha >4.58$.

## References

[1] Zhang J, Fang YP, Du WB, Cao XB (2011). Promotion of cooperation in aspiration-based spatial prisoner’s dilemma game. Physica A.

[2] Chen X, Fu F, Wang L. Could Feedback-Based Self-Learning Help Solve Networked Prisoner’s Dilemma? IEEE. 2009;1526–31.

[3] Zeng W, Li M, Feng N (2017). The effects of heterogeneous interaction and risk attitude adaptation on the evolution of cooperation. J Evol Econ.

[4] Liu Y, Chen X, Wang L, Li B, Zhang W, Wang H (2011). Aspiration-based learning promotes cooperation in spatial prisoner’s dilemma games. EPL-Europhys Lett.

[5] Perc M, Jordan JJ, Rand DG, Wang Z, Boccaletti S, Szolnoki A (2017). Statistical Physics of Human Cooperation. Phys Rep..

[6] Szolnoki A, Perc M (2010). Impact of critical mass on the evolution of cooperation in spatial public goods games. Phys Rev E.

[7] Chen X, Fu F, Wang L (2008). Promoting cooperation by local contribution under stochastic win-stay-lose-shift mechanism. Physica A.

[8] Perc M, Gomez-Gardenes J, Szolnoki A, Floria LM, Moreno Y (2013). Evolutionary dynamics of group interactions on structured populations: a review. J R Soc Interface.

[9] Liu Y, Chen X, Zhang L, Wang L, Perc M (2012). Win-stay-lose-learn promotes cooperation in the spatial prisoner’s dilemma game. PLOS ONE..

[10] Matsen FA, Nowak MA (2004). Win-stay, lose-shift in language learning from peers. Proc Natl Acad Sci USA.

[11] Chen X, Wang L (2008). Promotion of cooperation induced by appropriate payoff aspirations in a small-world networked game. Phys Rev E.

[12] Du J (2019). Redistribution promotes cooperation in spatial public goods games under aspiration dynamics. Appl Math Comput..

[13] Zhou L, Wu B, Vasconcelos VV, Wang L (2018). Simple property of heterogeneous aspiration dynamics: beyond weak selection. Phys Rev E.

[14] Peng Y, Wang X, Lu Q, Zeng Q, Wang B (2014). Effects of aspiration-induced adaptation and migration on the evolution of cooperation. Int J Mod Phys C..

[15] Lin H, Yang DP, Shuai JW (2011). Cooperation among mobile individuals with payoff expectations in the spatial prisoner’s dilemma game. Chaos Soliton Fract..

[16] Yang HX, Wu ZX, Wang BH (2010). Role of aspiration-induced migration in cooperation. Phys Rev E..

[17] Platkowski T, Bujnowski P (2009). Cooperation in aspiration-based n -person prisoner’s dilemmas. Phys Rev E..

[18] Platkowski T (2015). Aspiration-based full cooperation in finite systems of players. Appl Math Comput.

[19] Feng X, Wu B, Wang L (2018). Voluntary vaccination dilemma with evolving psychological perceptions. J Theor Biol.

[20] Tang C, Wang Y, Cao L, Li X, Yang Y (2013). Towards the role of social connectivity and aspiration level on evolutionary game. Eur Phys J B..

[21] Wu B, Zhou L (2018). Individualised aspiration dynamics: calculation by proofs. PLoS Comput Biol.

[22] Rong ZH, Zhao Q, Wu ZX, Zhou T, Chi KT (2016). Proper aspiration level promotes generous behavior in the spatial prisoner’s Dilemma game. Eur Phys J B.

[23] Wu ZX, Rong Z (2014). Boosting cooperation by involving extortion in spatial prisoner’s dilemma games. Phys Rev E..

[24] Wakano JY, Yamamura N (2001). A simple learning strategy that realizes robust cooperation better than pavlov in iterated prisoner’s dilemma. J Ethol..

[25] Platkowski T (2009). Enhanced cooperation in prisoner’s dilemma with aspiration. Appl Math Lett.

[26] Roca CP, Helbing D (2011). Emergence of social cohesion in a model society of greedy, mobile individuals. Proc Natl Acad Sci USA.

[27] Du J, Wu B, Wang L (2016). Aspiration dynamics and the sustainability of resources in the public goods dilemma. Phys Lett A.

[28] Zhang HF, Liu RR, Wang Z, Yang HX, Wang BH (2011). Aspiration-induced reconnection in spatial public goods game. EPL-Europhys Lett.

[29] Du J, Wu B, Wang L, Evolutionary Game Dynamics of Multi-Agent Cooperation Driven by Self-Learning. In: 9th Asian Control Conference.; 2013;1-6.

[30] Matjaz P, Wang Z (2010). Heterogeneous aspirations promote cooperation in the prisoner’s dlemma game. PLoS ONE..

[31] Li M, Jia CX, Liu RR, Wang BH (2013). Emergence of cooperation in spatial public goods game with conditional participation. Phys A.

[32] Du J, Wu B, Wang L (2015). Aspiration dynamics in structured population acts as if in a well-mixed one. Sci Rep.

[33] Li Z, Yang Z, Wu T, Wang L (2014). Aspiration-based partner switching boosts cooperation in social dilemmas. PLoS ONE.

[34] Xu K, Li K, Cong R, Wang L (2017). Cooperation guided by the coexistence of imitation dynamics and aspiration dynamics in structured populations. EPL-Europhys Lett.

[35] Liu X, He M, Kang Y, Pan Q (2016). Aspiration promotes cooperation in the prisoner’s dilemma game with the imitation rule. Phys Rev E.

[36] Chen W, Wu T, Li Z, Wang L, Coevolution of Aspirations and Cooperation in Spatial Prisoner’s Dilemma Game. J Stat Mech. 2015(P01032).

[37] Wu B, Gokhale CS, Wang L, Traulsen A (2012). How small are small mutation rates?. J Math Biol.

[38] Antal T, Nowak MA, Traulsen A (2009). Strategy abundance in 2 x 2 games for arbitrary mutation rates. J Theor Biol.

[39] Fudenberg D, Nowak MA, Taylor C, Imhof LA (2006). Evolutionary game dynamics in finite populations with strong selection and weak mutation. Theor Popul Biol.

[40] Fudenberg D, Imhof LA (2006). Imitation processes with small mutations. J Econ Theory.

[41] Willensdorfer M, Nowak MA (2005). Mutation in Evolutionary Games Can Increase Average Fitness At Equilibrium. J Theor Biol.

[42] Eriksson A, Lindgren K (2005). Cooperation driven by mutations in multi-person prisoner’s dilemma. J Theor Biol.

[43] Traulsen A, Claussen JC, Hauert C (2012). Stochastic differential equations for evolutionary dynamics with demographic noise and mutations. Phys Rev E.

[44] Tarnita CE, Antal T, Nowak MA (2009). Mutation cselection equilibrium in games with mixed strategies. J Theor Biol.

[45] Helbing D, Szolnoki A, Perc M, Szabo G (2010). Defector-accelerated cooperativeness and punishment in public goods games with mutations. Phys Rev E.

[46] Posch M, Pichler A, Sigmund K (1999). The efficiency of adapting aspiration levels. Proc Roy Soc B-Biol Sci.

[47] Posch M (1999). Win-stay, lose-shift strategies for repeated games - memory length, aspiration levels and noise. J Theor Biol.

[48] Du J, Wu B, Altrock PM, Wang L (2014). Aspiration dynamics of multi-player games in finite populations. J R Soc Interface.

[49] Wu T, Fu F, Wang L (2018). Coevolutionary dynamics of aspiration and strategy in spatial repeated public goods games. New J Phys.

[50] Tanimoto J (2013). Difference of reciprocity effect in two coevolutionary models of presumed two-player and multiplayer games. Phys Rev E.

[51] Tanimoto J, Sagara H (2007). Relationship between dilemma occurrence and the existence of a weakly dominant strategy in a two-player symmetric game. Bio Syst.

[52] Wang Z, Kokubo S, Jusup M, Tanimoto J (2015). Universal scaling for the dilemma strength in evolutionary games. Phys Life Rev.

[53] Ito H, Tanimoto J (2018). Scaling the phase-planes of social dilemma strengths shows game-class changes in the five rules governing the evolution of cooperation. Roy Soc Open Sci.

[54] Ito H, Tanimoto J (2020). Dynamic utility: the sixth reciprocity mechanism for the evolution of cooperation. Roy Soc Open Sci.

[55] Tanimoto J (2019). Evolutionary games with sociophysics: analysis of taffic flow and epidemics.

[56] Tanimoto Jun (2015). Fundamentals of evolutionary game theory and its applications.

[57] Alesina A, Angeletos G, Fairness and Redistribution. Am Econ Rev 2005.

[58] Fernandez R, Levy G (2008). Diversity and redistribution. J Public Econ.

[59] Xie W, Ho B, Meier S, Zhou X (2017). Rank reversal aversion inhibits redistribution across societies. Nat Hum Behav.

[60] Li Y, Zhang J, Perc M (2018). Effects of compassion on the evolution of cooperation in spatial social dilemmas. Appl Math Comput.

[61] Du J, Wang B (2018). Evolution of global cooperation in multi-level threshold public goods games with income redistribution. Front Phys.

[62] Antal T, Traulsen A, Ohtsuki H, Tarnita CE, Nowak MA (2009). Mutation-selection equilibrium in games with multiple strategies. J Theor Biol..

[63] Kaiping GA, Jacobs GS, Cox SJ, Sluckin TJ (2014). Nonequivalence of Updating Rules in Evolutionary Games Under High Mutation Rates. Phys Rev E.

[64] Allen B, Traulsen A, Tarnita CE, Nowak MA (2012). How mutation affects evolutionary games on graphs. J Theor Biol.

[65] Yakushkina T, Saakian DB (2018). New Versions of Evolutionary Models with Lethal Mutations. Phys A.

[66] Wu B, Traulsen A, Gokhale CS (2013). Dynamic properties of evolutionary multi-player games in finite populations. Games.

[67] Gokhale CS, Traulsen A (2010). Evolutionary games in the multiverse. Proc Natl Acad Sci USA.

[68] Arnoldt H, Timme M, Grosskinsky S (2012). Frequency-dependent fitness induces multistability in coevolutionary dynamics. J R Soc Interface.

[69] Souza MO, Pacheco JM, Santos FC (2009). Evolution of cooperation under N-person snowdrift games. J Theor Biol.

[70] Doebeli M, Hauert C (2005). Models of Cooperation Based On the Prisoner’s Dilemma and the Snowdrift Game. Ecol Lett.

[71] Du J, Tang L (2018). Evolution of Global Contribution in Multi-Level Threshold Public Goods Games with Insurance Compensation. J Stat Mech.

[72] Wang X, Chen X, Gao J, Wang L (2013). Reputation-based mutual selection rule promotes cooperation in spatial threshold public goods games. Chaos Soliton Fract..

[73] Chen XJ, Wang L (2010). Effects of cost threshold and noise in spatial snowdrift games with fixed multi-person interactions. EPL-Europhys Lett..

[74] Wang XJ, Xia K (2019). Extended average abundance function of multi-player snowdrift evolutionary game under aspiration driven rule. Syst Eng Theory Pract.

[75] Wu B, Altrock PM, Wang L, Traulsen A (2010). Universality of weak selection. Phys Rev E.

